# Recent genomic heritage in Scotland

**DOI:** 10.1186/s12864-015-1605-2

**Published:** 2015-06-06

**Authors:** Carmen Amador, Jennifer Huffman, Holly Trochet, Archie Campbell, David Porteous, Generation Scotland, James F Wilson, Nick Hastie, Veronique Vitart, Caroline Hayward, Pau Navarro, Chris S Haley

**Affiliations:** MRC IGMM, University of Edinburgh, Edinburgh, EH4 2XU UK; Centre for Population Health Sciences, University of Edinburgh, Edinburgh, EH8 9AG UK; Roslin Institute and Royal (Dick) School of Veterinary Studies, University of Edinburgh, Edinburgh, EH25 9RG UK

**Keywords:** Generation Scotland, Principal component analysis, Genetic ancestry, Admixture, Rare variants, Population structure

## Abstract

**Background:**

The Generation Scotland Scottish Family Health Study (GS:SFHS) includes 23,960 participants from across Scotland with records for many health-related traits and environmental covariates. Genotypes at ~700 K SNPs are currently available for 10,000 participants. The cohort was designed as a resource for genetic and health related research and the study of complex traits. In this study we developed a suite of analyses to disentangle the genomic differentiation within GS:SFHS individuals to describe and optimise the sample and methods for future analyses.

**Results:**

We combined the genotypic information of GS:SFHS with 1092 individuals from the 1000 Genomes project and estimated their genomic relationships. Then, we performed Principal Component Analyses of the resulting relationships to investigate the genomic origin of different groups. We characterised two groups of individuals: those with a few sparse rare markers in the genome, and those with several large rare haplotypes which might represent relatively recent exogenous ancestors. We identified some individuals with likely Italian ancestry and a group with some potential African/Asian ancestry. An analysis of homozygosity in the GS:SFHS sample revealed a very similar pattern to other European populations. We also identified an individual carrying a chromosome 1 uniparental disomy. We found evidence of local geographic stratification within the population having impact on the genomic structure.

**Conclusions:**

These findings illuminate the history of the Scottish population and have implications for further analyses such as the study of the contributions of common and rare variants to trait heritabilities and the evaluation of genomic and phenotypic prediction of disease.

**Electronic supplementary material:**

The online version of this article (doi:10.1186/s12864-015-1605-2) contains supplementary material, which is available to authorized users.

## Background

The Generation Scotland Scottish Family Health Study (GS:SFHS) is a family-based genetic epidemiology study which includes 23,960 participants in ~7,000 family groups from across Scotland. Participants were recruited by letter of invitation from general practitioner lists to provide a representative sampling of the population. There was no selection on the basis of medical status or history. All were interviewed and clinically assessed for a wide range of health-related traits and environmental covariates, and linked also to routine health records [1, 2]. Ten thousand of these participants, of whom ~6,000 are not known to be directly related, also have genotypic information for ~700 K SNPs. The cohort was designed as a resource for genetic and health-related research. So far, projects based on this cohort are underway to study the genetics of depression, the prediction of cardiovascular disease risk, or the role of specific genes in asthma, bronchitis and emphysema, but the potential uses of GS:SFHS are much wider (http://www.generationscotland.org/). The GS:SFHS cohort is a family-based study, and a fair proportion of individuals in the population will be related, some unknowingly. This structure will allow the shared variation between individuals within families to be disentangled into its genetic and environmental components and should facilitate accurate estimations of heritability. In addition, genome-wide association studies to be carried using the GS:SFHS cohort will need to adequately account for population substructure. Without proper correction, false-positive results can arise as a consequence of stratification differences, particularly between cases and controls, because of allele frequency differences or differences in LD patterns [3–5]. As genomic relationships are an integral part of the statistical methods used to unravel or utilise trait variation and affect their performance [5, 6] a detailed genomic description of the structure of GS:SFHS is a prerequisite to the application of these mapping and prediction methods. Furthermore, such description will shed some light on the demographic history, the existence and characterization of hidden ancestral structure and the amount and origin of the variability in the population.

Our aim was to provide an accurate genomic description of the GS:SFHS cohort, which could reflect as well the history of Scottish population. For that purpose, we developed several genomic approaches using the information of ~700 K SNPs in the individuals of the cohort. Our objectives were to: 1) place GS:SFHS in the context of other human populations by exploring their genetic variation and establish the ancestry of participants, 2) identify the extent and the origin of rare haplotypes in GS:SFHS individuals, 3) explore genetic differentiation within GS:SFHS, 4) analyse homozygosity in the GS:SFHS cohort, 5) identify the extent of geographic differentiation within the sample, and 6) apply the knowledge gained from these studies to identify a core set of samples to select the most appropriate for different future analyses.

These exhaustive analyses will not only reflect an accurate picture of the demographic history of Scotland, but also will have implications for our further studies using the GS:SFHS cohort in terms of the genomic differentiation found among its individuals, (e.g., introgression detected, homozygosity, etc.) which will help select the most appropriate groups of individuals for each future analysis. These studies could include a detailed analysis of the contributions of common and rare variants to trait heritabilities, haplotype mapping, and evaluation of genomic and phenotypic prediction of disease.

## Results

### Placing Generation Scotland in a global genomic context

**Fig. 1:**
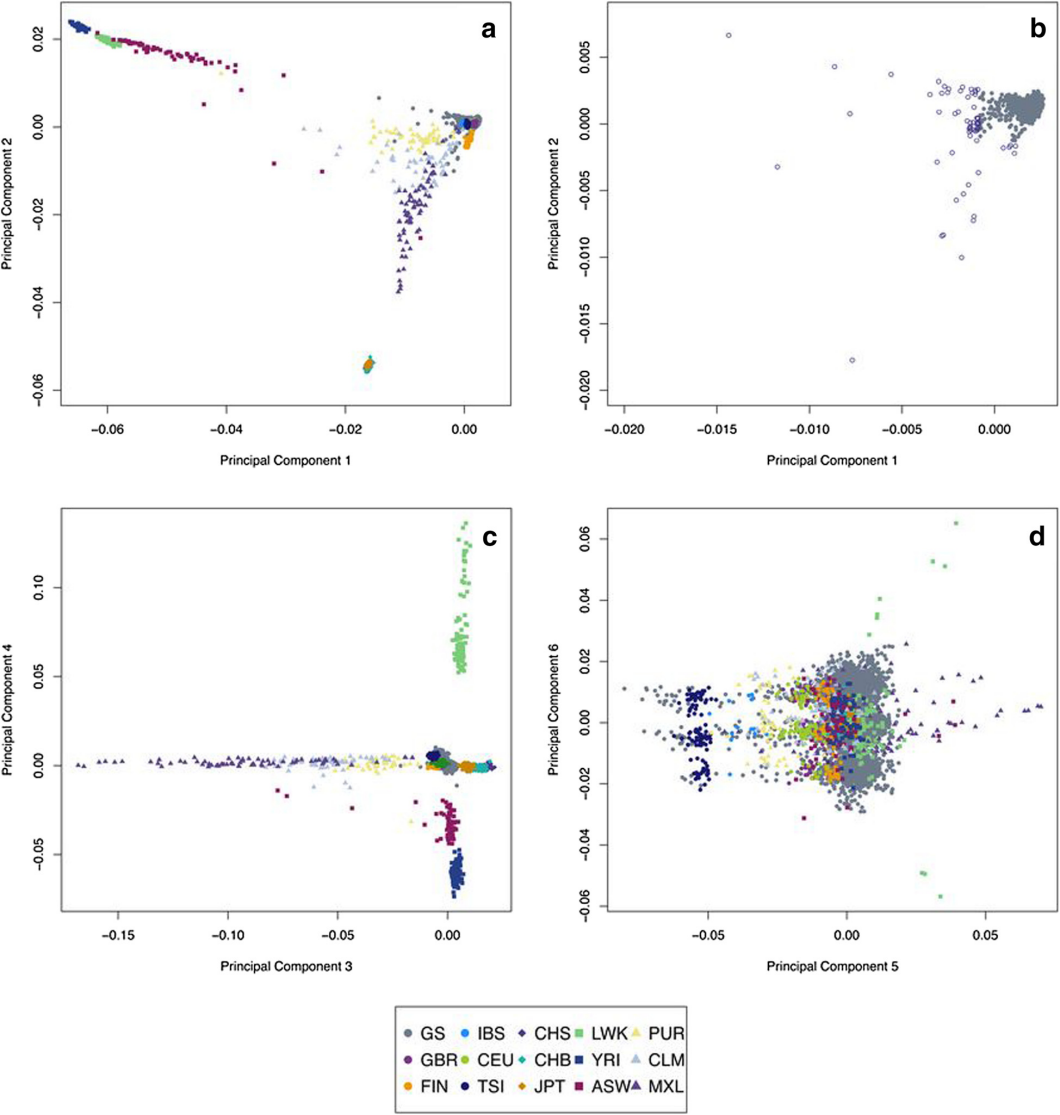
Results of the PCA in the GS + 1 kG data set. **a** Values for PC1 and PC2 in GS + 1 kG individuals; **b** Values for PC1 and PC2 only in GS:SFHS individuals (open circles were defined as outliers); **c** Values for PC3 and PC4 in GS + 1 kG individuals; **d** Values for PC5 and PC6 in GS + 1 kG individuals

We performed a set of analyses to place GS:SFHS into context of a sample of other global populations. First, we merged GS:SFHS with the data of 1092 individuals from the 1000 Genomes Project (Table 1), and we calculated a Genomic Relationship Matrix (GRM) from the marker data of the resulting data set (GS + 1 kG). Then, we performed a Principal Component Analysis (PCA) of the GRM. Results of the PCA of the GRM in the population GS + 1 kG are presented in Fig. 1: Fig. 1a shows a plot of the first two PCs (those with the largest eigenvalues); Fig. 1b shows a detail of the same figure including only the GS:SFHS individuals, Fig. 1c shows a plot of the third and fourth PCs and Fig. 1d shows a plot of the fifth and sixth PCs. Plots for PCs 7–20 are shown in Additional file 1: Figure S1.

**Table 1:** Origin, location, number of individuals and given values for latitude and longitude for the different populations in the GS + 1 kG data set

Code	Origin	Location	N. ind
GS:SFHS	Europe	Scotland	9,889
ASW	Africa	African ancestry individuals in SW US	61
CEU	Europe	Utah residents with N. and W. European ancestry	85
CHB	Asia	Han Chinese in Beijing	97
CHS	Asia	Han Chinese South	100
CLM	America	Colombian in Medellin, Colombia	60
FIN	Europe	Finnish individuals in Finland	93
GBR	Europe	British individuals in England and Scotland	89
IBS	Europe	Iberian populations in Spain	14
JPT	Asia	Japanese individuals in Tokyo, Japan	89
LWK	Africa	Luhya individuals in Webuye, Kenya	97
MXL	America	Mexican ancestry individuals in LA California	66
PUR	America	Puerto Rican in Puerto Rico	55
TSI	Europe	Tuscan individuals in Tuscany, Italia	98
YRI	Africa	Yoruba individuals in Ibadan, Nigeria	88
Total			10,981

The first and second principal component separated the African (different coloured squares), East Asian (different coloured rhomboids) and European (different coloured circles) populations (Fig. 1a). American populations (different coloured triangles) spread over the plot between the three main groups (Africans, East Asians and Europeans). African ancestry individuals living in the US (ASW, red squares) were more spread towards the European populations than the two populations from Africa (Luhya and Yoruba) which remained separated in a more consistent group, owing to the history of European contributions to ASW. The East Asian populations formed a tight group clearly independent of the rest. When looking at the plot of GS:SFHS within this graph (Fig. 1b), it can be noticed that a very small but obvious proportion of GS:SFHS individuals leaned towards the African population, whereas a similarly small but also obvious proportion were closer to the East Asian populations. This analysis suggests that some GS:SFHS participants have mixed ethnic backgrounds. For example, 64 individuals have values for PC1 or PC2 more than 6 standard deviations away from the mean. They are plotted as open blue circles in Fig. 1b.

If we look at further PCs, we observed, as shown in Fig. 1, that the third and fourth PCs reflected some variation within the Mexican population from LA (MXL) and African origin populations, respectively (Fig. 1c). For both eigenvectors GS:SFHS individuals were located together with the 1 kG European populations.

The fifth PC separated the Italian population from Tuscany from the rest of the populations (Fig. 1d). However, some individuals from GS:SFHS clustered with this Italian group consistent with them having some similar ancestry.

In the sixth PC of the GRM of GS + 1 kG, a different pattern appears. For this PC the individuals from most of the populations are separated in three clear clusters, mostly detected in GS:SFHS because of its larger sample size. The SNPs causing this clustering are located in chromosome 8, in the 8p23.1 region. A known common inversion polymorphism is located in this region [7, 8] suggesting that it might be the cause of the clustering pattern we observe.

### Generation Scotland in a European genomic context

**Fig. 2:**
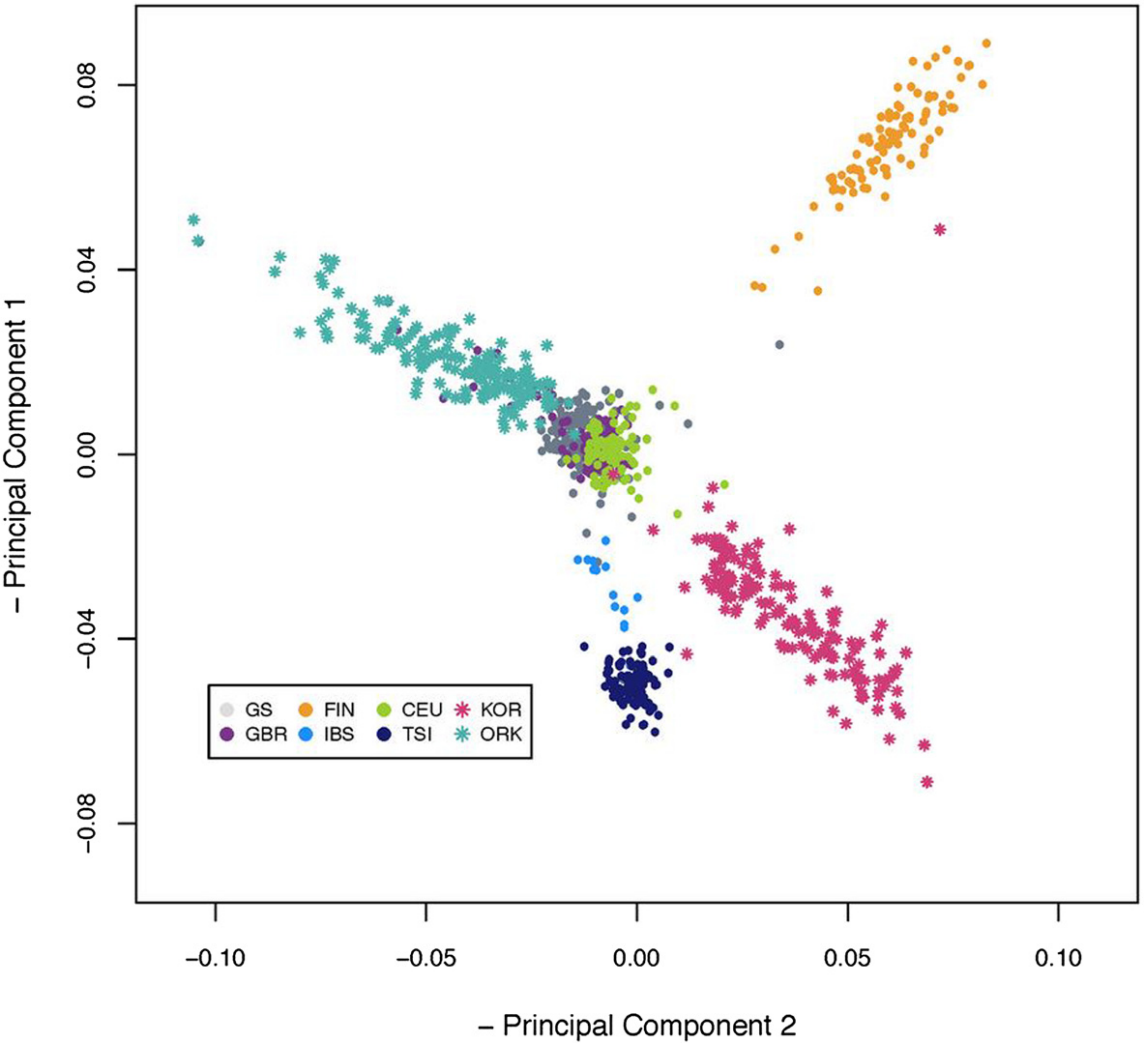
Results of the PCA in the GS + European data set. Values for PC1 and PC2 in Generation Scotland and the other European samples

To place Generation Scotland into a European context we performed a PCA of a random sample of 200 individuals of GS:SFHS together with the European samples of 1 kG (CEU, GBR, FIN, IBS and TSI) and 150 Orcadian individuals (ORK) and 150 Croatian individuals (KOR). Results are shown in Fig. 2 for PC 1 and PC 2. The plot shows a distribution of populations similar to a map of Europe (note the axes are inverted) as in [9], where the Italian (TSI), Iberian (IBE) and Croatian (KOR) populations are located at the bottom of the plot (south), the Finnish (FIN) and Orcadian (ORK) populations in the top (north) and the rest mostly in the centre. GS:SFHS is located consistently between Orkney and the British (GBP) population.

### Genetic structure due to rare alleles

To thoroughly explore the ancestries of the individuals in the GS:SFHS cohort and gain insight into the potential origins of the outliers we analysed the patterns of allele sharing described by genomic relationship coefficients. It is clear from the formulation of the standard estimator (1, see Methods and Additional file 1: Figure S2) that shared rare alleles can have a substantial influence on estimates in the GRM. To measure the influence of the rare alleles in the relationships between individuals of GS:SFHS we used three scores: [1] an *individual marker score* that measures how each marker contributes in an individual to its relationships with the rest of the population; [2] a *pair marker score* that measures how each marker affects the relationship between a particular pair of individuals; and [3] a *rarity score* to measure the overall amount of rare variants that an individual has. The details of these calculations are provided in the Methods section.

**Fig. 3:**
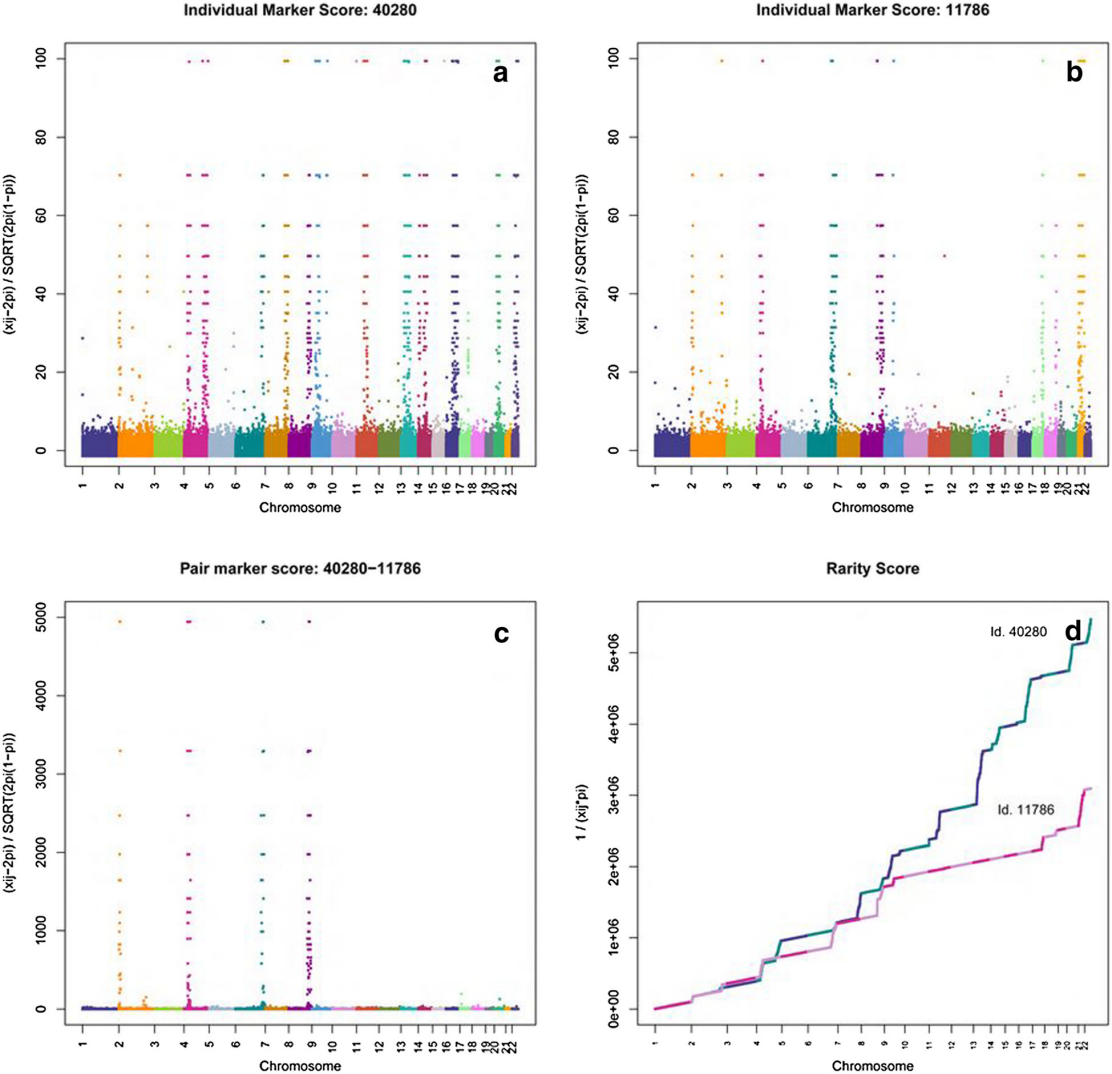
*Score* values of individuals 40,280 and 11,786. **a** *Individual marker score* of individual 40,280; **b** *Individual marker score* of individual 11,786; **c** *Pair marker score* of individuals 40,280 and 11,786; **d** *Rarity scores* of individual 40,280 and 11,786

Table 2 demonstrates the impact of the inclusion of rare alleles in our data, by showing the values for the genomic relationship coefficients truncating the SNP data at different allele frequencies between selected extreme examples of pairs of individuals that are not related to each other according to the pedigree (lying in the more extreme positions towards African populations in Fig. 1b). A comparison between the values of relationship coefficients obtained when using different allele frequency thresholds for the whole population are presented in Additional file 1: Figure S3, indicating that only for a small minority of relationships does the inclusion of rare alleles make a difference. The genomic relationship coefficients obtained using all the markers for the pairs of individuals in Table 2 show values between 0.17 and 0.45, which are around the values expected for third and first degree relatives respectively, and are unlikely to arise between unrelated individuals. When we re-estimated the relationships between the same pairs of individuals, excluding SNPs with rare alleles, these relationships decreased to lower values (between 0.008 and 0.08) as expected between unrelated or distantly related individuals. To explore the impact of rare alleles in individual relationships across the genome we selected the first pair of individuals in Table 2 to analyse further their relationship. Results are plotted in Fig. 3. Figure 3a and b show the *individual marker scores* [1] of each individual respectively. Figure 3c shows their *pair marker score* [2] and Figure 3d shows the *rarity scores* for both individuals [3].

**Table 2:** Genomic relationship coefficients between several pairs of individuals using different thresholds for the computation of the GRM

Ind. 1	Ind. 2	GRM_ALL_	GRM_>1 %_	GRM_>5 %_	GRM_<1 %_	GRM_<5 %_
40280	11786	0.453	0.080	0.075	4.776	2.638
132098	30436	0.341	0.062	0.049	3.580	2.025
67527	30436	0.263	0.038	0.029	2.870	1.612
145349	30436	0.187	0.011	0.006	2.217	1.226
147185	30436	0.455	0.053	0.038	5.120	2.863
147185	132098	0.272	0.038	0.027	2.999	1.691
147185	67527	0.230	0.026	0.021	2.602	1.440
147185	145349	0.181	0.014	0.012	2.123	1.162
147185	34327	0.175	0.017	0.013	2.010	1.116
147185	9025	0.180	0.008	0.007	2.177	1.184
147185	118411	0.242	0.030	0.022	2.702	1.518
114918	30436	0.195	0.011	0.006	2.315	1.281
108361	147185	0.195	0.021	0.015	2.216	1.237
153784	30436	0.200	0.043	0.033	2.007	1.158
153784	145349	0.219	0.026	0.024	2.467	1.354
153784	147185	0.458	0.043	0.034	5.295	2.918
153784	108361	0.195	0.017	0.014	2.257	1.240
40280	30436	0.271	0.024	0.017	3.133	1.734
40280	147185	0.176	0.008	0.003	2.132	1.178
62626	147185	0.173	0.015	0.012	2.015	1.110

The peaks for the *individual marker scores* in each of the graphs (Fig. 3a and b) represent areas where the individuals carry some rare alleles (p ≤ 0.005). The SNPs causing the inflated relationship are represented by the *pair marker score* in Fig. 3c. The rare alleles that both individuals share are located in chromosomes 2, 4, 6 and 9 which demonstrate common peaks in Fig. 3a and b. Figure 3d shows the *rarity score* of individual 40280 plotted as a cumulative score, where number and magnitude of changes in the slope, as well as the total *rarity score* value, are greater than for individual 11786. The remaining pairs in Table 2 showed a similar pattern of sharing when plotting their *individual* and *pair scores*.

The same graphs are plotted in Additional file 1: Figure S4 for two randomly selected GS:SFHS individuals. The number of peaks is lower than in the previous plot, with a few sparse high values, and no peaks in the *pair score*. Both individuals’ *rarity score* are considerably smaller than the previous shown in Fig. 3.

**Fig. 4:**
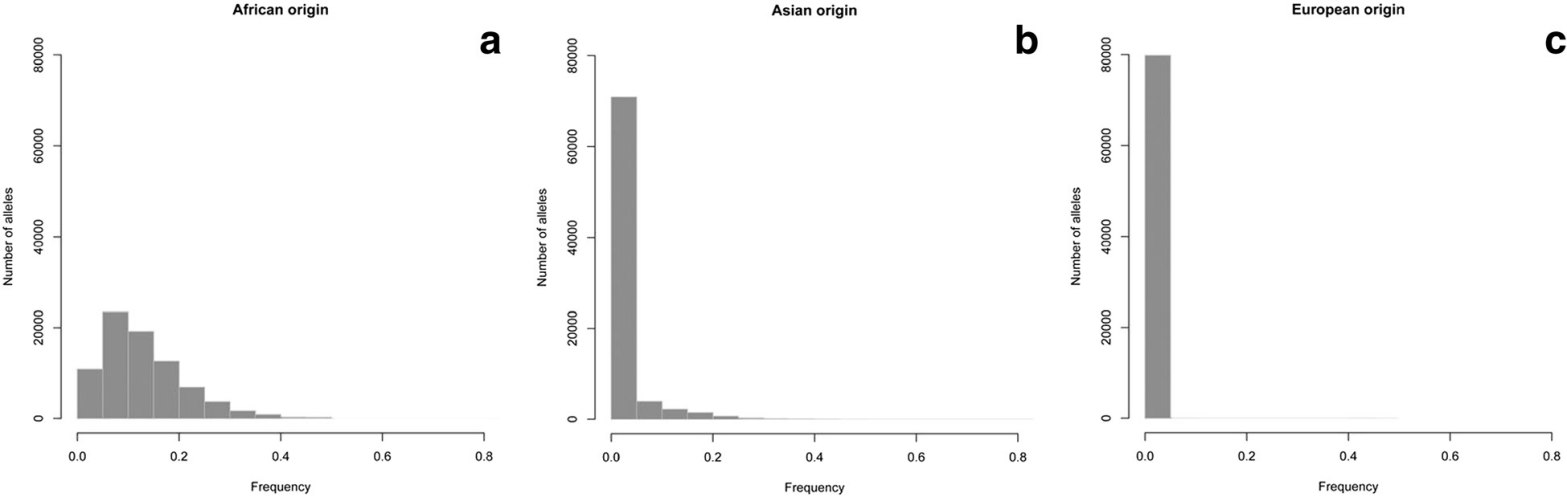
Origin of rare alleles in GS:SFHS. Frequencies for rare Generation Scotland alleles (p ≤ 0.0004) in the **a** African, **b** Asian and **c** European populations of the 1000 Genomes data set

In the case of an individual carrying an exogenous allele, it is expected that it will increase the *rarity score* because it would be at low frequency (see Eq. 4 in Methods). We analysed the origin of these low frequency alleles by selecting markers in the population contributing to the *rarity score* with a value larger than 2,500 (i.e., *p*_*i*_ ≤ 0.0004 or 8 or less rare alleles in total in the whole GS:SFHS population) and plotting the frequencies for those alleles in the African, East Asian and European populations of the 1 kG data set. The results are shown in Fig. 4. The analysis of the probable origin of the rare alleles shows that while the frequency of these alleles is nearly always close to zero in the European populations their values in East Asian and particularly African populations are clearly higher, suggesting an African origin for the majority of the cases.

Additional file 1: Figure S5 shows a histogram for the *rarity score* values [3] for all the individuals in GS:SFHS. The mean value for *rarity scores* was 1,071,738 ± 259,736. Using the rarity scores in windows of 50 SNPs, we calculated the number of *rare peaks* in all the individuals (see Genetic structure due to rare alleles in Methods). The mean number of peaks per individual is 5.6 and the mean total coverage of peaks is 3.3 Mb. Those individuals with a total coverage of peaks larger than the mean plus three times the standard deviation were considered outliers (74 individuals). Table 3 shows the mean, maximum and minimum number of peaks in outliers and non-outliers. Results show that individuals classified as non-outliers have a mean of 5.3 *rare peaks* over their genome whereas outliers have on average 46.5. In addition, the total coverage of the peaks is very different between both groups, with the mean coverage in the non-outliers being 2.3 Mb (0.07 % of the genome) and 136.7 Mb for the outliers (4 % of the genome). Histograms showing the distribution of the percentage of rare genome and the number of peaks in the non-outliers and the outliers are shown in Additional file 1: Figure S6.

**Table 3:** Distribution of the peaks detected using the rarity scores in windows of 50 SNPs in the two groups of individuals (outliers and non-outliers)

	Non-Outliers	
	Number of peaks per individual	Total coverage of peaks per individual (Mb)
Mean	5.3	2.3
Max	118	50
Min	0	0
	Outliers	
	Number of peaks per individual	Total coverage of peaks per individual (Mb)
Mean	46.5	136.7
Max	185	800
Min	11	51

Table shows mean, maximum and minimum number of peaks and total peak size per individual

These values can help characterise the two groups of individuals. The non-outliers have a few sparse and small peaks in their genome potentially reflecting a point mutation (in most of the cases the window score is due to one single marker at a low frequency). On the other hand, the individuals defined as outliers can reflect a different kind of ancestry: the size of the peaks suggests that these individuals have a more admixed background, due to having a recent foreign ancestor.

We checked the self-reported origin of the 74 individuals detected as outliers and although the amount of non-disclosed information is slightly higher than in the whole data set, the recorded origin of their grandparents did not indicate that they came from outside the UK.

### Individual ancestries using ADMIXTURE

To provide additional evidence of our results, we analysed a subset of markers in approximate linkage equilibrium with the software ADMIXTURE [10] to estimate the proportion of ancestral populations in GS + 1 kG individuals assuming 3 ancestral populations, i.e., African, Asian and European (K = 3). Additional file 1: Figure S7 shows the proportion of each origin in the individuals of 1 kG and GS:SFHS. The correlation between the estimated proportion of African origin and the percentage of the genome covered by peaks was 0.94 showing that our estimates correspond well with percentage of African genes in the individuals (Additional file 1: Figure S8).

### Areas of high linkage disequilibrium in Generation Scotland

**Fig. 5:**
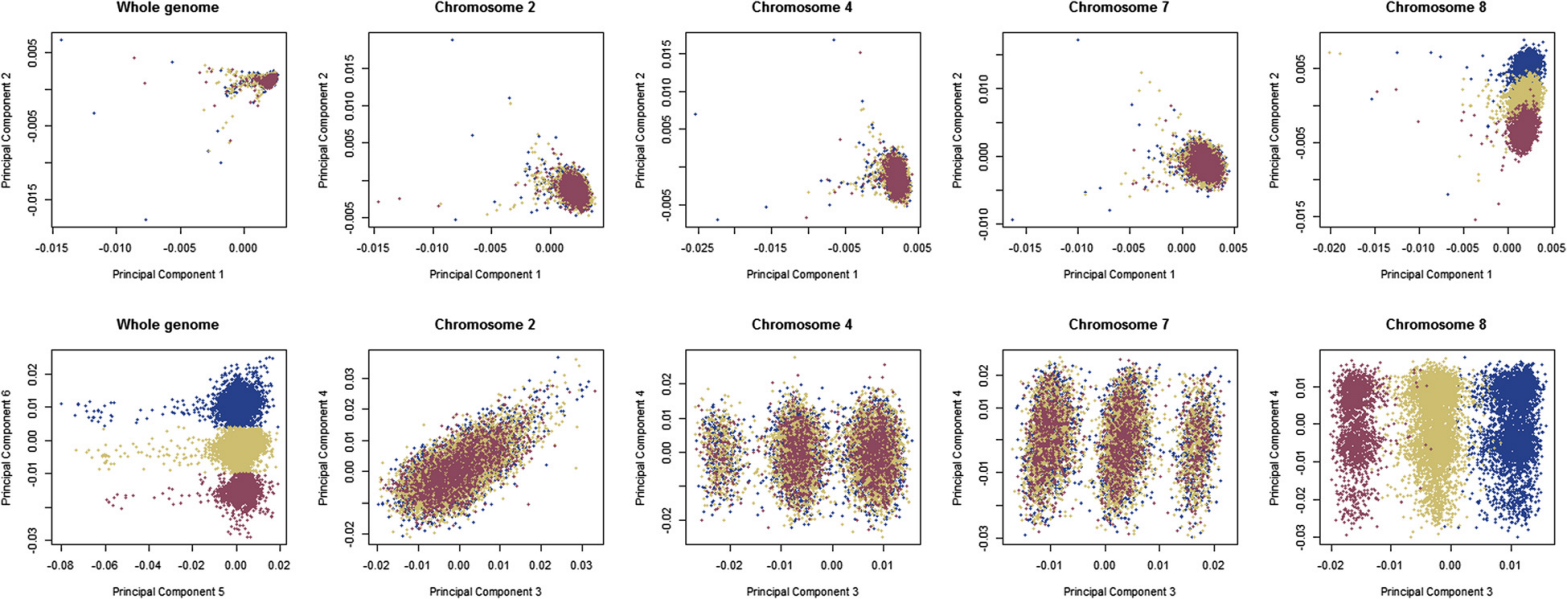
Selected results from chromosomal PCA. Location of GS:SFHS individuals (whole genome and different chromosomes analyses) for PC 1 and 2 (*upper row*) and several PCs showing a distinct pattern (*lower row*). The colours show the correspondence between the groups shown in PC 5 and 6 when using the whole genome, and those obtained when analysing only chromosome 8 for PC 2 and 3

Areas in high linkage disequilibrium can drive a lot of variation in the PCs, as we observed with the inversion in chromosome 8 and MHC in chromosome 6, which is responsible of the clustering observed for several PCs [11, 12]. In order to detect other large regions in LD which could represent inversions, conserved regions, etc., we performed a PCA per chromosome in the GS + 1 kG population. The most extreme examples of clustering are shown in Fig. 5 and the complete plots for PC 1 to 20 per chromosome are shown in Additional file 1: Figure S8.

The plots of PC three and/or four showed a three-cluster pattern for some of the chromosomes. In some cases the three groups were clearly distinguishable (chromosomes 4, 7, 8, 15), and in some others the pattern was created by combining both eigenvectors together or it became less clear (chromosomes 6, 10, 11, 12, 19, 21, 22). Sometimes two different clustering patterns could be seen in both the third and fourth PCs (chromosome 8, 12, 15) (Additional file 1: Figure S9).

The SNPs responsible for the PCs clustering were chromosome-wide for the first and second PCs for all chromosomes, showing a very similar pattern (except for chromosome 6 and 8) and to the clustering of individuals obtained for the original genome-wide PCA (Fig. 1b). This shows that the differences driven by the first two eigenvectors between all the individuals of GS:SFHS and the rest of the origins are genome-wide and consistent between chromosomes, representing differences across populations.

A summary of a literature review aiming to identify plausible causes of the other observed three-cluster patterns in the different PCs can be found in Additional file 2: Table S1, including genes located in the identified regions. These areas appear to be regions of high linkage disequilibrium, so that individuals inherited long haplotypes and that caused the clustering pattern observed. This linkage disequilibrium could be due to different reasons, such as selection, including past selective sweeps around new alleles/mutations, or long inversions, or chance. In chromosome 2, the SNPs causing the observed clustering for PC3 (Additional file 1: Figure S9) is likely due to the *Lactase* gene which has been shown to display clines of variation due to selection [11]. In addition, the SNPs in the region 8p23.1 (chromosome 8 inversion) caused the pattern in PC 2 and 3 of chromosome 8; and in chromosome 6 the MHC was identified as the cause the clustering in PCs 1 to 6. This area has previously been suggested to display selection pressure associated variation [11]. MHC associated SNPs showed a lot of influence in the whole genome PCA, accounting for the variation explained by several PCs. Some centromeric areas were also detected as responsible for the variance explained in several PCs. With respect of the other three group patterns detected, we found several genes located in the areas involved (Additional file 2: Table S1) that could be responsible for the clustering. In the case of chromosome 15 PC3, only one gene is located in the responsible area, ALDH1A2 (Aldehyde dehydrogenase 1 family, member A2).

### Runs of homozygosity in Generation Scotland

In order to explore the patterns of homozygosity in GS:SFHS, we estimated the length and location of homozygous segments in each individual of the cohort (Additional file 1: Figure S10). Additional file 1: Figure S10a shows the distribution of the number of segments per individual and Additional file 1: Figure S10b the distribution of the total length of the homozygous segments per individual. Additional file 1: Figure S10c shows the number of ROHs compared to the total length of ROHs and Additional file 1: Figure S10d the proportion of individuals with one or more ROHs of a given length. The distribution of the homozygous segments in GS:SFHS is similar to those described in other studies in European populations [13, 14]. When comparing the results with those obtained for the controls of the Scottish Colon Cancer Study (SOCCS, 984 subjects from Scotland not known to have colon cancer) we observed that the percentage of large ROHs described in GS:SFHS is slightly higher than that found in SOCCS, which is also a mainland Scottish population [13].

The analysis also revealed that one female individual carried a completely homozygous chromosome 1 suggesting an instance of uniparental disomy. Genomic information from other related individuals pointed out that she had inherited two copies of the paternal chromosome 1.

To evaluate the possible association between ROH and the *rarity score* in the GS:SFHS individuals, we performed a linear regression between the total length of homozygous segments or the number of runs per individual and the values for the *rarity score*. The *rarity score* showed a negative correlation with both total length and number of ROH (Additional file 1: Figure S11) suggesting that more homozygous individuals would have less rare haplotypes.

### Population structure within Generation Scotland

**Fig. 6:**
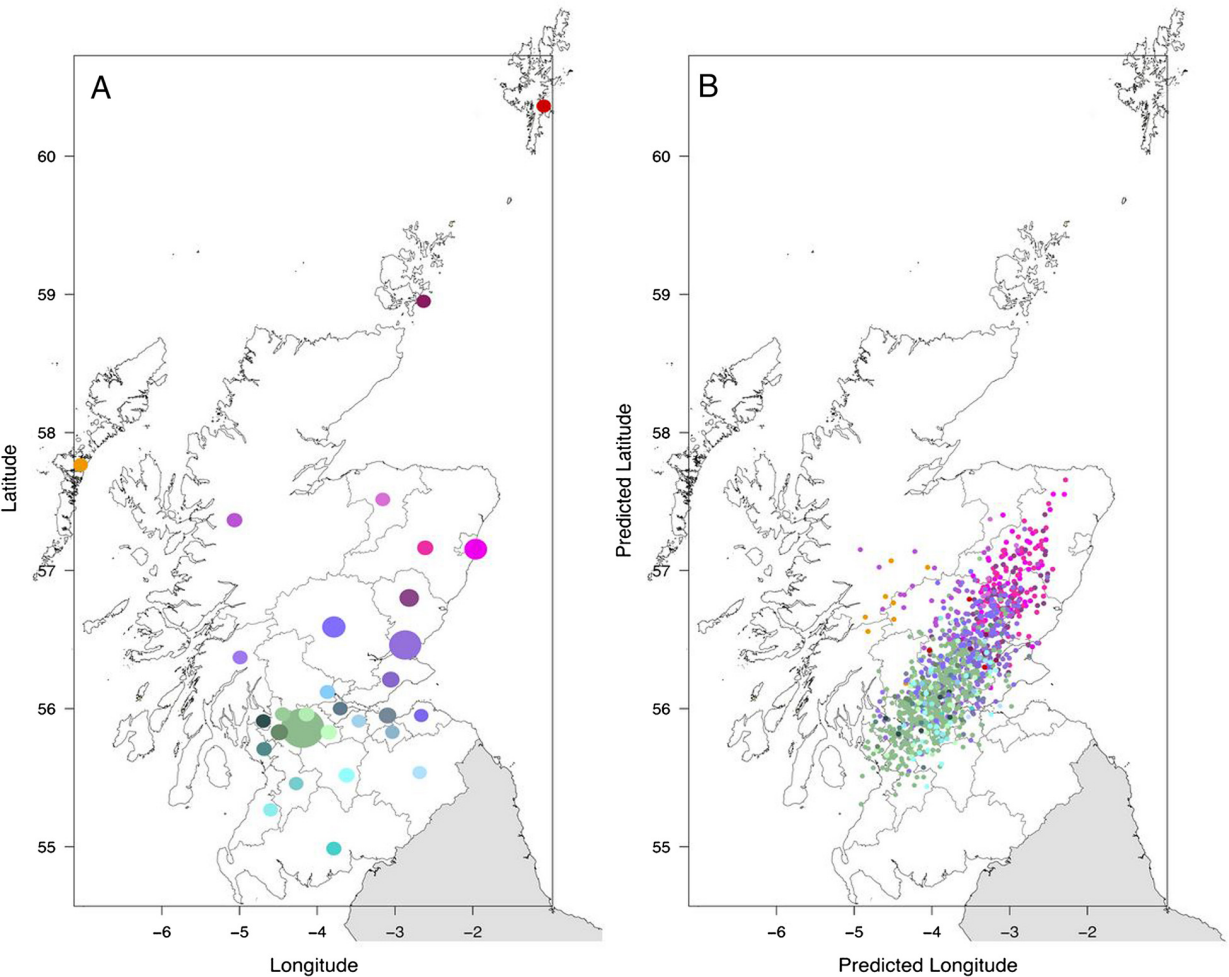
Locations and predictions within Scotland. **a** Real location of the 31 different origins of the GS:SFHS individuals. **b** Predicted latitude and longitude of the individuals using the genomic principal components

To unravel any possible link between the genomic differentiation and geographic stratification in the sample we created a pruned subset of SNPs in approximate linkage equilibrium with each other in order to capture the structure that reflects geographic origin. We removed all the markers from chromosome 6 (to remove the effect of the *Major Histocompatibility Complex*, MHC) and markers in chromosome 8 located in the region 8 p23.1, since both areas have been proven to have a big impact on the PCA [9, 11, 12]. Also, to remove familiar structure, we removed related individuals from the data set (i.e., one individual in each pair with a genomic relationship coefficient larger than 0.025), individuals detected as having Italian origin, and those with mixed ethnic backgrounds. The resulting data set consisted in 6739 unrelated individuals. Results are presented in Additional file 1: Figure S12 for PC one to twenty. In order to evaluate the impact of origin within Scotland on the PCs of the GS:SFHS participants, we assigned a value for latitude and longitude according to their birthplaces and calculated the regression of the eigenvectors on the geographic coordinates (see Table 4). The results are shown in Table 5. Most of the PCs (except for 4, 6, 9, 12 and 15) showed a significant association with geographic origin. In all of these cases the longitude was significant, and the latitude was significant for some PCs. Longitude explained 11 % of the variance within PC 1, and both latitude and longitude jointly explained 16 % and 9 % of PC 2 and PC 3 respectively. The significant models for the rest of PCs (4 to 20) explained a very small proportion of the variance (*R*^*2*^ ≤ 0.01). This shows that geography has an impact on the genomic variation of GS:SFHS, especially on that contributing to principal components 1 to 3. To further investigate this influence we used the recorded origin of grandparents, in order to obtain a more accurate picture of the individual’s “genetic origin”, so we selected a subset of individuals consisting in those with four grandparents coming from the same area (e.g., four grandparents from Aberdeen, or four from Dundee). We used this new subset (consisting on 1113 individuals) to calculate a new regression of the principal components on the geographic coordinates. The results are shown in Table 6. Most of the PCs were significant but in this case latitude and longitude explained 21 %, 31 % and 21 % of PCs 1, 2 and 3 respectively. A plot of the first three principal components is shown in Additional file 1: Figure S13. We used the values of the principal components to predict the “genomic” latitude and longitude of each individual, i.e., to predict where a given individual comes from “genomically”. Figure 6 shows the results of the predicted values. Figure 6a shows the observed latitude and longitude of “genetic origin” of the individuals according to their grandparents’ origin. The size of the points in Fig. 6a reflects the number of individuals with origin from a given region. Figure 6b shows the results of the predicted values of latitude and longitude using PCs 1 to 20. The colours show the observed origin using the same coding as Fig. 6a. The results show that the prediction of latitude and longitude corresponds well with the observed origin. The correlation between the real and predicted latitude was 0.67 and the correlation between the real and predicted longitude was 0.62.

**Table 4:** Areas in Scotland, number of individuals in the cohort born in each of the areas, number of individuals with the four grandparents coming from that area, and values of latitude and longitude used for each of the areas in the regression analyses

Area		N ind.	N 4GPs	Lat.	Lon.
1	Aberdeen City	470	78	57.15	-2.09
2	Aberdeenshire	100	85	57.16	-2.72
3	Angus	290	66	56.80	-2.92
4	Argyll & Bute	48	6	56.37	-5.03
5	Clackmannanshire	0	0	56.12	-3.55
6	Dumfries & Galloway	44	7	54.99	-3.86
7	Dundee City	1016	202	56.46	-2.97
8	East Ayrshire	23	5	55.46	-4.33
9	East Dunbartonshire	80	4	55.96	-4.20
10	East Lothian	11	1	55.95	-2.77
11	Edinburgh City	189	21	55.95	-3.19
12	Western Isles	18	8	57.76	-7.02
13	Falkirk	34	4	56.00	-3.78
14	Fife	184	26	56.21	-3.15
15	Glasgow City	1644	414	55.86	-4.25
16	Highland	77	29	57.36	-5.10
17	Inverclyde	28	6	55.91	-4.74
18	Midlothian	14	0	55.83	-3.13
19	Moray	36	7	57.51	-3.25
20	North Ayrshire	60	8	55.71	-4.73
21	North Lanarkshire	117	24	55.83	-3.92
22	Orkney Islands	8	5	58.94	-2.74
23	Perth & Kinross	512	49	56.59	-3.86
24	Renfrewshire	167	12	55.83	-4.54
25	Scottish Borders	22	4	55.54	-2.79
26	Shetland Islands	8	3	60.35	-1.24
27	South Ayrshire	31	7	55.27	-4.65
28	South Lanarkshire	110	20	55.52	-3.70
29	Stirling	67	5	56.12	-3.94
30	West Dunbartonshire	66	7	55.96	-4.50
31	West Lothian	1	0	55.91	-3.55
-	Not disclosed	1338	-	NA	NA

**Table 5:** Results of the multiple linear regressions between the PC and the values of latitude and longitude in 6739 unrelated individuals of GS:SFHS

Analysis	R^2^	*p*-value		Latitude	Longitude
PC1	0.1092	4.20E-139	***	***	***
PC2	0.1632	6.81E-214	***	***	***
PC3	0.0936	2.47E-118	***	***	***
PC4	0.0010	6.26E-02		*	
PC5	0.0070	4.21E-09	***	***	***
PC6	0.0003	3.88E-01			
PC7	0.0077	6.13E-10	***		***
PC8	0.0035	6.51E-05	***		***
PC9	0.0002	5.15E-01			
PC10	0.0144	4.35E-18	***	**	***
PC11	0.0024	1.39E-03	**		*
PC12	0.0008	1.09E-01			
PC13	0.0093	6.74E-12	***	**	***
PC14	0.0016	1.09E-02	*	*	**
PC15	0.0001	8.28E-01			
PC16	0.0033	1.04E-04	***		***
PC17	0.0023	1.91E-03	**	***	*
PC18	0.0018	7.54E-03	**	*	
PC19	0.0033	1.01E-04	***		***
PC20	0.0046	3.35E-06	***		***

Signif: ***p ≤ 0.001, ** p ≤ 0.01, *p ≤ 0.05

**Table 6:** Results of the multiple linear regressions between the PC and the values of latitude and longitude of the grandparents in 1113 individuals of GS:SFHS

Analysis	R^2^	*p*-value		Latitude	Longitude
PC1	0.2077	7.42E-57	***	**	***
PC2	0.3085	1.24E-89	***	***	**
PC3	0.2056	3.26E-56	***	***	*
PC4	0.0100	3.68E-03	**	***	
PC5	0.0301	4.34E-08	***	***	***
PC6	0.0100	3.85E-03	**	**	**
PC7	0.0247	9.27E-07	***		***
PC8	0.0085	9.00E-03	**		**
PC9	0.0076	1.47E-02	*		**
PC10	0.0552	2.06E-14	***	***	***
PC11	0.0037	1.26E-01			
PC12	0.0055	4.65E-02	*	*	
PC13	0.0082	1.06E-02	*		**
PC14	0.0057	4.30E-02	*	*	*
PC15	0.0017	3.93E-01			
PC16	0.0147	2.73E-04	***		***
PC17	0.0148	2.62E-04	***	***	***
PC18	0.0047	7.29E-02			
PC19	0.0045	8.16E-02			
PC20	0.0102	3.44E-03	**		**

Signif: ***p ≤ 0.001, **p ≤ 0.01, *p ≤ 0.05

### Impact of population structure and geographic location on health-related traits

To explore the impact of the region of “genomic” origin (defined using predicted longitude and latitude) and the area of residence of the individuals in a variety of health-related traits, we used two different mixed linear models. We explored the variance explained by a similarity matrix constructed using the values of predicted latitude and longitude (i.e., the values in the matrix represent “genomic” proximity between individuals). We fitted a mixed linear model as in [15] including a GRM and the similarity matrix (Geo), estimated using GCTA [16] the proportion of the phenotypic variance captured by each of these matrices, and compared them when including or not the current area of residence as a fixed effect. The results of the analysis are shown in Additional file 1: Table S3 for body mass index (BMI), fat, waist-to-hip ratio (WHR) and high density lipoprotein (HDL). In all scenarios, the matrix Geo explained a small but significant amount of the trait variance and this remained the case when current area of residence was included in the model.

We also explored the effects of sex, age, age^2^, predicted latitude and longitude (as covariates) and the current area of residence when fitted in a mixed linear model with a GRM. Results are shown in Additional file 1: Table S4 for the four traits. The results show that longitude is significant for fat and WHR with the effect being reduced values further east in both traits. Some of the areas of residence show also significant effects.

## Discussion

Historic and demographic events leave their signatures in the genomes of populations. These genomic marks allow us to track relationships, reconstruct introgression events and find different patterns in the populations that can be linked with structural variation or reflect areas in high linkage disequilibrium. This study provides a detailed picture of the genetic structure within the GS:SFHS cohort and some historic demographic events that have shaped the genomes of its individuals.

Our analyses showed some expected patterns together with some valuable information regarding GS:SFHS individuals. The first and second principal components of the genomic relationship matrix in the GS + 1 kG population separated the European from the African and East Asian populations respectively. They also showed that a few individuals from the Scottish population have a variable pattern of genes from different ancestries, with part of this group of individuals having more than a 4 % of non-European origin according to the rare allele analysis. These non-European regions are too large to be explained as random mutations or genotyping errors. It is widely accepted that the Eurasian populations were originated from a single “out of Africa” event [17], and that the African populations are genetically more diverse than European and Asian samples because of this bottleneck [18]. This means that rare alleles in Europe may be common in Africa. Gabriel et al. [19] estimated that half of the human genome exists in blocks of 44 kb or larger in European populations with a maximum of 173 kb blocks. Even considering that the blocks in GS:SFHS could be larger, the length of the regions described by Gabriel et al. [19] still suggests that the “out of Africa” bottleneck cannot explain haplotypes of up to 7 Mb with rare variants, indicating that the outliers have a relatively recent ancestor likely coming from an African population. Also, Wall and Pritchard [20] reviewed several studies of haplotype blocks in human populations and showed the correlation between the recombination rate and the length of the regions. We checked the recombination rate in some of the regions that we detected and it was close or larger to the 1.2 cM/Mb expectation [21], which indicates that the long non-European regions we uncover are not the result of regions of low recombination. These individuals should probably be removed from the data set for subsequent analyses, such as common and rare variants contribution to heritability, haplotype mapping, genomic prediction of disease, etc., otherwise allele frequencies differences could bias results.

The rest of the population appears to be more homogeneous when analysing the results of the PCA and the rare allele analysis. They show an average proportion of peaks of 0.07 % and a more compact clustering pattern. This 0.07 % of peaks is likely to reflect, at least partially, point mutations that occurred in recent generations. Some of these mutations could also be genotyping errors, but we cannot differentiate them.

The pattern of rare alleles detected is highly influenced by the SNPs genotyped so it is likely that the ascertainment bias of the chip has an impact on our results. Whereas with a different chip different variants might have been detected as coming from other populations, we do not expect spurious results arising because of this in the rare allele analysis.

The correlation between the estimated proportion of African origin using ADMIXTURE and the percentage of the genome covered by peaks was high (0.94) showing that our estimates correspond well with percentage of African genes in the individuals (Additional file 1: Figure S8). Our estimates of Asian ancestry seem to be underestimated when compared to the ADMIXTURE results.

Despite the homogeneity of the majority of the population (i.e., excluding the outliers), it would probably still be useful to correct for some subjacent population structure in further analyses by including the eigenvectors as covariates in the models in future association studies or heritability estimations [5].

Regarding the homozygosity patterns, the distribution of the number of homozygous segments and their length in GS:SFHS was similar to that described in previous studies in other European populations (Figure S10) [13, 14]. However, the proportion of individuals carrying large ROHs was slightly higher than found previously in the SOCCS population [13], particularly for those ROH between 2.5 and 5 Mb (frequency in GS:SFHS 50 %, frequency in SOCCS <30 %). *Rarity scores* showed a negative correlation with the number and total length of ROH as expected, since the more admixed individuals are in general less homozygous.

We identified a group of individuals with Italian ancestry in GS:SFHS through their similarity with the Tuscan individuals in the 1000 Genomes populations (Fig. 1d). This is consistent with a large influx of Italian immigration to Scotland at the end of XIX century and beginning of XX century (1880–1920 Scotland’s Census). The Italians in GS:SFHS appear as a distinct group from the rest of the population in the PCA, but they do not seem to differ much when analysing their rare alleles. The values of their *rarity scores* do not appear as outliers in the population. This implies that there are not big differences in their allele frequencies when comparing to the rest of GS:SFHS (consistently with the fact that they are also a European population). As the architecture of allelic frequencies is the same, we can assume that the underlying architecture of complex traits will be very similar as well. We consider that these individuals could be included for most of the future analyses if the population structure is accounted for using whole genome marker information. Regarding the rest of the European populations, GS:SFHS individuals are located between Britain and Orcadian populations as expected and further away from more southern populations.

The genome-wide information in GS:SFHS allowed us to discover some other variation. The inversion in chromosome 8 is one of the largest polymorphic inversions found in humans (~4.5 Mb) with a frequency of ~20–50 % in European populations, ~59 % in the Yoruba and ~12–27 % in Asians [7, 8]. It can be predicted from genotypes [7, 22] and it was previously identified via principal components by Zou et al. [12]. This information suggests that selection against crossovers in the region maintains the linkage disequilibrium pattern [12]. Previous studies that detected this inversion, were capable of detecting a smaller (900 Kb) inversion in chromosome 17 (17q21) with a 20 % frequency in Europeans [12, 22, 23]. We did not find any signal for any of the SNPs in any of the principal components in chromosome 17 pointing at that area.

The chromosome 8 inversion proves that a linkage disequilibrium region can be detected using genotypes and principal component analysis. The similar clustering patterns in the rest of the chromosomes discovered through the chromosomal PCA suggest regions of between 260 Kb (15q21.3) and ~7.5 Mb (6p22.3-p21.32) in high linkage disequilibrium. This long haplotype patterns could be informative of selective sweeps in the past that have not been broken down by recombination. For some of these areas expected results pointed out the variation due to selection of the lactase gene (chromosome 2) or the *Major Histocompatibility Complex* in chromosome 6 [11]. The other areas identified contain several genes, and it is difficult to pinpoint more accurately the specific gene or region that causes the observed clustering pattern, which could suggest some selective pressure over it.

As others, we have shown that the geographic differentiation between populations from different origins (Africa, East Asia, and Europe) is detected in the PCA but we have also found that the geographic origin within Scotland has a significant impact on the principal components. We were able to predict with relative high accuracy the origin of the individuals from their genomic data. The small number of samples from the islands (Western Isles, Orkney and Shetland), and hence their limited influence in the PCA, made that prediction of the origin of these individuals was somehow poorer than that of individuals from regions with more genotyped participants. Most of the families participating in the study were recruited in big cities (which usually gather people from different origins creating a non-homogeneous sample) and the information from the grandparents’ origin is often not complete. This could affect the results obtained from our origin prediction analysis, for which prediction was more efficient at separating individuals from the South-West from those of the North-East of Scotland (separating mostly Glasgow, Dundee and Aberdeen).

When we explored the impact of predicted location based on information from the regions of origin of the individual’s grandparents (i.e., “genomic” origin) and current place of residence on four health-related traits (BMI, fat, WHR and HDL), we observed that genomic origin had an impact on these traits. In the four cases, the similarity matrix based on geographic proximity significantly captured some phenotypic variance, irrespective of including current region of residence in the model. Also, when examining the effects of predicted latitude and predicted longitude we observed that longitude had a significant effect for fat and WHR but latitude did not, when both variables were jointly fitted. It has to be noticed that the distribution of the samples over Scotland makes latitude and longitude correlated to each other (*r* = 0.63). After adding to the model the current region of residence of the individuals, the predicted longitude of genomic origin was still significant, and similar results were obtained when we replaced current region of residence with region of birth (results not shown). Although these associations with genomic origin as predicted by grandparental birthplace and are not removed by adjustment for either an individual’s place of birth or current place of residence, it is possible that they are the result of undetected stratification in the sample. This could include, for example, persistence of cultural transmission of lifestyle or dietary habits associated with grandparental origins.

## Conclusions

All these results will have an important impact over future studies that use the GS:SFHS cohort and correspond well with known Scottish demographic history. We have performed a thorough analysis of genomic data in GS:SFHS, applying standard methods such as the use of PCA, but also inspecting the results of such analyses in depth We have discovered some individuals with mixed ancestries that should be removed for future studies (e.g., GWAS) and we have characterised some other that can remain in the cohort.

## Methods

### Data set

The data were obtained from the Generation Scotland: Scottish Family Health Study (GS:SFHS) [1]. Ethical approval for the study was given by the NHS Tayside committee on research ethics (reference 05/s1401/89). Governance of the study, including public engagement, protocol development and access arrangements, was overseen by an independent advisory board, established by the Scottish government. Research participants gave consent to allow both academic and commercial research.

Individuals were genotyped with the Illumina OMNIExpress chip (706,786 SNPs). We used GenABEL version 1.7-6 [24] and PLINK version 1.07 [25] to exclude SNPs that had a missingness >2 % and a Hardy-Weinberg Equilibrium test P < 10^−6^. Duplicate samples, individuals with gender discrepancies and those with more than 2 % missing genotypes were also removed. After this quality control, the data set consisted in 9889 individuals (4085 males and 5804 females) with multiple degrees of kinship (~6000 non related), genotyped for 646,127 SNP spread over the 22 autosomes. The recorded information about the locations of origin of these individuals is shown in Additional file 1: Figure S14.

In order to investigate ancestry, the GS:SFHS data set was merged to the data of 1092 individuals of different origins (Table 1) from the 1000 Genomes Project (1 kG) [26]. The resulting data set (GS + 1 kG) had 10,981 individuals genotyped for 635,190 markers common to all populations spread over the 22 autosomes.

To further investigate the relationships of GS:SFHS with other European populations, another data set was created. 200 individuals (randomly selected) from Generation Scotland and the samples with European origin in the 1 kG Project (CEU, FIN, GBR, IBS and TSI, see Table 1) were merged with two new populations. 150 individuals from a Croatian population from the Dalmatian island of Korcula (KOR) [27] and 150 individuals from another Scottish population from the Orkney Islands (ORK) [13]. Korcula received ethical approval from the Ethics Committee of the Medical School, University of Split and the NHS Lothian (South East Scotland Research Ethics Committee). The ORCADES study (referred as ORK), received ethical approval from the NHS Orkney Research Ethics Committee and North of Scotland Research Ethics Committee. All participants signed informed consent prior to participation.

This data set (GS + EU) consisted in 879 individuals genotyped for 97,648 markers in approximate linkage equilibrium and with minor allele frequencies (MAF) larger than 0.01.

### Genomic relationship matrix

Relationships were estimated from the genotyped SNP data and summarised into a genomic relationship matrix (GRM) using GCTA version 1.13 [16]. In the first instance, we used all autosomal SNP data that had passed quality control (regardless of MAF). In order to investigate the effect of the allele frequency spectrum of the SNPs used to compute the GRM on relationship estimation, we computed five extra GRMs with different SNP MAF thresholds in the merged population GS + 1 kG. The number of markers corresponding to the thresholds applied is shown in Additional file 1: Table S2. We also computed a new GRM in the GS:SFHS population, after removing areas in high linkage disequilibrium (LD). To do this, we removed all SNPs from chromosome 6 (to remove the influence of *Major Histocompatibility Complex*) and those located in area 8p23.1 (to remove the influence of a large polymorphic inversion). Then, we generated a pruned subset of markers in approximate linkage equilibrium by using the default *indep-pairwise* command in PLINK [25]. We also excluded related individuals using the *grm-cutoff* command in GCTA [16] with a value of 0.025 (i.e., removing iteratively one of a pair of individuals with a relationship coefficient larger than 0.025). An extra GRM was also computed in the GS + EU population. These GRMs were used as input for the Principal Component Analyses described in the following section that aim to assign ancestral origins to GS:SFHS participants, and to establish the nature of information on relatedness conveyed by markers of different allele frequencies. In addition, to establish the contribution of each chromosome to the observed structure, twenty two extra GRMs (chromosomal GRMs) were calculated i.e., one for each autosome, using only the markers in one chromosome (from 1 to 22) without excluding any marker that had passed quality control irrespective of its frequency in the GS + 1 kG merged population.

### Principal component analyses

Principal component analysis (PCA) is a widely used method to convert a set of observations for different (and possible related) variables into values of linearly uncorrelated variables called principal components or eigenvectors. If we apply it to the GRM, we can use it to determine, control and correct for population structure, as usually done in genome-wide association studies. Resulting clusters will reflect individuals that group together because of higher genomic similarities [5]. We carried out an eigenanalysis of the GRM of the GS:SFHS population. In order to put into context the variation observed in GS:SFHS (and any potential outliers in the population, as per [28]), we used the populations in the 1 kG data as outgroups, and carried out a PCA of the GS + 1 kG population (of the GRM computed using all markers), and a PCA of the GS + EU population. In addition we performed 22 eigenanalysis using the 22 chromosomal GRM. All analyses were conducted in ACTA version 0.9 [29]. We calculated and used in further analyses the first 20 eigenvectors or principal components (PCs) per analysis.

For some of the analyses, to locate the specific areas of the genome causing the different clustering patterns in the different PCs, genome-wide association analyses of the principal components were performed. We tested for association and estimated the effect of each SNP on the values observed for the eigenvectors of interest. In each analysis, the values of each principal component were analysed as a phenotype in a linear model fitting each SNP in turn and including sex, age and age^2^ as covariates using the linear function in PLINK v1.07 [17].

### Genetic structure due to rare alleles

The values in the GRM provide an estimation of the genomic relationships between individuals irrespective of their pedigree relationships. GCTA uses a formula which measures the allele sharing between each pair of individuals, weighted by the frequency of the markers. We have observed that this method obtains accurate relationships due to common alleles, but it inflates some relationships due to rare allele sharing. The GRM elements represent identity by descent between two different individuals *j* and *k* calculated as:

1 $$ \frac{1}{N}{\displaystyle {\sum}_i\frac{\left({x}_{ij}-2{p}_i\right)\left({x}_{ik}-2{p}_i\right)}{2{p}_i\left(1-{p}_i\right)}},j\ne k $$

[15]

Where *N* is the total number of markers, *x*_*ij*_ is the genotype of individual *j* at marker *i* (coded as 0, 1 or 2, representing the number of copies of the less frequent allele) and *p*_*i*_ is the minor allele frequency (MAF) of marker *i* in the population.

The formula weights the allele sharing by the allele frequency in the population. This means that some pairs of individuals sharing rare alleles can show an unrealistic inflated value of their genomic relationship coefficient. This happens frequently in individuals known to be closely related according to pedigree records (such as siblings or parent–child relationships) because it is more likely in these relationships to share rare alleles, but it sometimes happens with individuals that are totally unrelated according to the pedigree, but may share alleles due to a common ethnic ancestral origin. We intended to explore further these relationships by selecting those unrelated pairs of individuals with a genomic relationship coefficient larger than expected. We picked those pairs of individuals with an estimated large genomic relationship coefficient (0.17-0.50) when using all available markers but a very low (suggesting “unrelated”) coefficient (0–0.08) when markers with a rare allele were removed and only markers with common alleles used to estimate relationships. For those individuals, we calculated an *individual marker score* as the individual contribution to the GRM formula per marker as:

2 $$ \frac{\left({x}_{ij}-2{p}_i\right)}{\sqrt{2{p}_i\left(1-{p}_i\right)}} $$

And for each pair of individuals *j* and *k*, their *pair marker score*, as the relationship per marker between them as (i.e., the *pair marker score* is the multiplication of both *individual marker scores*):

3 $$ \frac{\left({x}_{ij}-2{p}_i\right)\left({x}_{ik}-2{p}_i\right)}{2{p}_i\left(1-{p}_i\right)} $$

In addition, to measure the overall amount of rare variants in all individuals of the cohort, a *rarity score* per individual was calculated. For those markers with at least one rare allele (i.e., *x*_*ij*_ ≠ 0) we summed the inverse of the allele doses multiplied by the allele frequency as:

4 $$ {\displaystyle \sum_{i=1}^N\frac{1}{x_{ij}{p}_i}} $$

This makes markers with a low MAF in GS:SFHS (smaller values of *p*_*i*_) contribute to a greater extent to the *rarity score*. The rarest allele of those markers with low MAF in GS:SFHS are more likely to have come from a different population, and we hypothesise that they have come (“been introgressed”) into the Scottish population from another, distinct, population. To further analyse the structure of rare areas in the genome of GS:SFHS individuals, we estimated the number of *rare peaks* per individual as follows: we divided each chromosome in non-overlapping windows of 50 SNPs and we calculated the mean *rarity score* in the window as the mean of all the *rarity score* values of the all the markers in a window. We defined as *rare peaks* those windows with a mean *rarity score* larger than 50 (e.g., those where the harmonic mean allele frequency across the 50 loci in the window is less than approximately 0.02). We assumed that windows with a mean *rarity score* larger than 50 separated by less than 10 other windows (i.e., less than 500 SNP) belonged to the same *rare peak* that also included all the in-between windows. We tested several window lengths and thresholds and obtained similar results for all the analyses.

### Estimation of individual ancestries using ADMIXTURE

We used the software ADMIXTURE [10] to estimate the proportion of ancestral populations in GS + 1 kG individuals. We reduced the number of markers in the GS + 1 kG dataset to remove linkage disequilibrium. A subset of markers (81,981 SNPs) was generated by using the default *indep-pairwise* command in PLINK. Then, we estimated the proportion African, Asian and European origin (K = 3) in all individuals of GS + 1 kG.

### Runs of homozygosity

To analyse the pattern of homozygosity in the GS:SFHS cohort, a screen for runs of homozygosity (ROH) was performed using PLINK v1.07 [25] with the same parameters as in McQuillan et al. [13]. The program slides a moving a window of 5,000 Kb (with a minimum of 50 SNPs) and locates and estimates the length of stretches of homozygous genotypes across the genome. The analysis allows one heterozygote genotype (allowing for example for genotyping errors) and 5 missing genotypes per window for the region to still be considered homozygous. The maximum gap between consecutive SNPs to be considered in the same ROH was 100 Kb.

### Influence of the geographic origin in the genomics of GS:SFHS

To evaluate the correlation between geographic origin of the individuals and the principal components in the analyses performed, we calculated the regression between the different values of the PCs and the values of latitude and longitude assigned to the origin of the individuals. We used the first 20 whole-genome PCs to develop two different sets of analyses. Individuals were given a value of latitude and longitude according to their birth place, as shown in Table 4, and the multiple regressions of each PCs over both values were calculated. We also extracted a subset of individuals with four grandparents having the same origin (1113 individuals). Fig. 6a shows the distribution of the origins over a map of Scotland. The size of the points represents the number of individuals with four grandparents from each area. We then performed a multiple linear regression between the values of the first 20 PCs in the 1113 individuals and the latitude and longitude of the grandparents’ origins. For these analyses the individuals detected as having Italian origin and those with mixed ethnic backgrounds detected in the GS + 1 kG PCA were removed in order to avoid bias in the geographic component. We also used the values of the PCs to predict latitude and longitude in these individuals by fitting a linear model regressing latitude or longitude against the values of the PCs for each individual. All regression analyses were performed using R version 3.0.1. [30].

### Impact of population structure and geographic location on health related traits

We also explored the impact of the predicted genomic origin of the individuals and their area of residence on four health-related traits: body mass index (BMI), fat, waist-to-hip ratio (WHR) and high density lipoprotein (HDL). We used the values of the PCs to predict latitude and longitude of genomic origin in the unrelated individuals subset using the model detailed in the previous section (*Influence of the geographic origin in the genomics of GS:SFHS*). Then, for each trait, we used GCTA [16] to fit two different mixed linear models.

Model 1:

$$ y=\mathbf{X}\upbeta +\mathrm{g}+\upvarepsilon, $$

where *y* is a vector of observed phenotypes, β is an vector of fixed effects and **X** its design matrix, g is a vector of additive genetic effects with assumed distribution: g ~ *N* (0, GRMσ_g_^2^). GRM is a genomic relationship matrix calculated using all autosomal SNPs with a MAF > 0.05.

We estimated the effects of the covariates: sex, age, age^2^, predicted latitude, predicted longitude and current area of residence (based on the different Postal Codes, see regions in Additional file 1: Table S4) and explored if the origin of the individuals estimated from their genomic data (i.e., the predicted geographic coordinates, that represent an average of the origin of the four grandparents) has an impact on the traits.

In Model 2:

$$ y=\mathbf{X}\upbeta +\mathrm{g}+{s}_{\mathrm{geo}}+\upvarepsilon, $$

we also included s_geo_ with assumed distribution: s_geo_ ~ *N* (0, GEOσ_s_^2^). GEO is a similarity matrix based derived from the predicted latitude and predicted longitude. It contains values between 0 and 1 reflecting (geographic) proximity between individuals according to their “genomic” origin. All values in the diagonal are equal to 1.

In this model we fitted sex, age, age^2^ as covariates and compared the estimates of σ_g_^2^ (V(GRM)) and σ_s_^2^ (V(GEO)) with those obtained fitting the same covariates and including also current place of residence in order to explore if there is a change in the variance explained by those models.

## Additional files

Additional file 1:
**Additional Figures S1-S14 and Additional Tables S2-S4.**
Additional file 2: Table S1. Results for regions responsible of the PCs forming three-cluster patterns detected in the GWAS analysis: Chromosome, number of the PC showing the pattern, length of the area (in base pairs), chromosomal region where it is located and genes located in them (including complete name and known functions).
