# Supplementary material for: Recent genomic heritage in Scotland
Source: BMC Genomics. 2015 Jun 6;16(1):437. doi: 10.1186/s12864-015-1605-2 (PMC4458001; doi:10.1186/s12864-015-1605-2)
Supplement: Additional file 1: — Additional Figures S1-S14 and Additional Tables S2-S4. [file 12864_2015_1605_MOESM1_ESM.pdf]

Fig S1

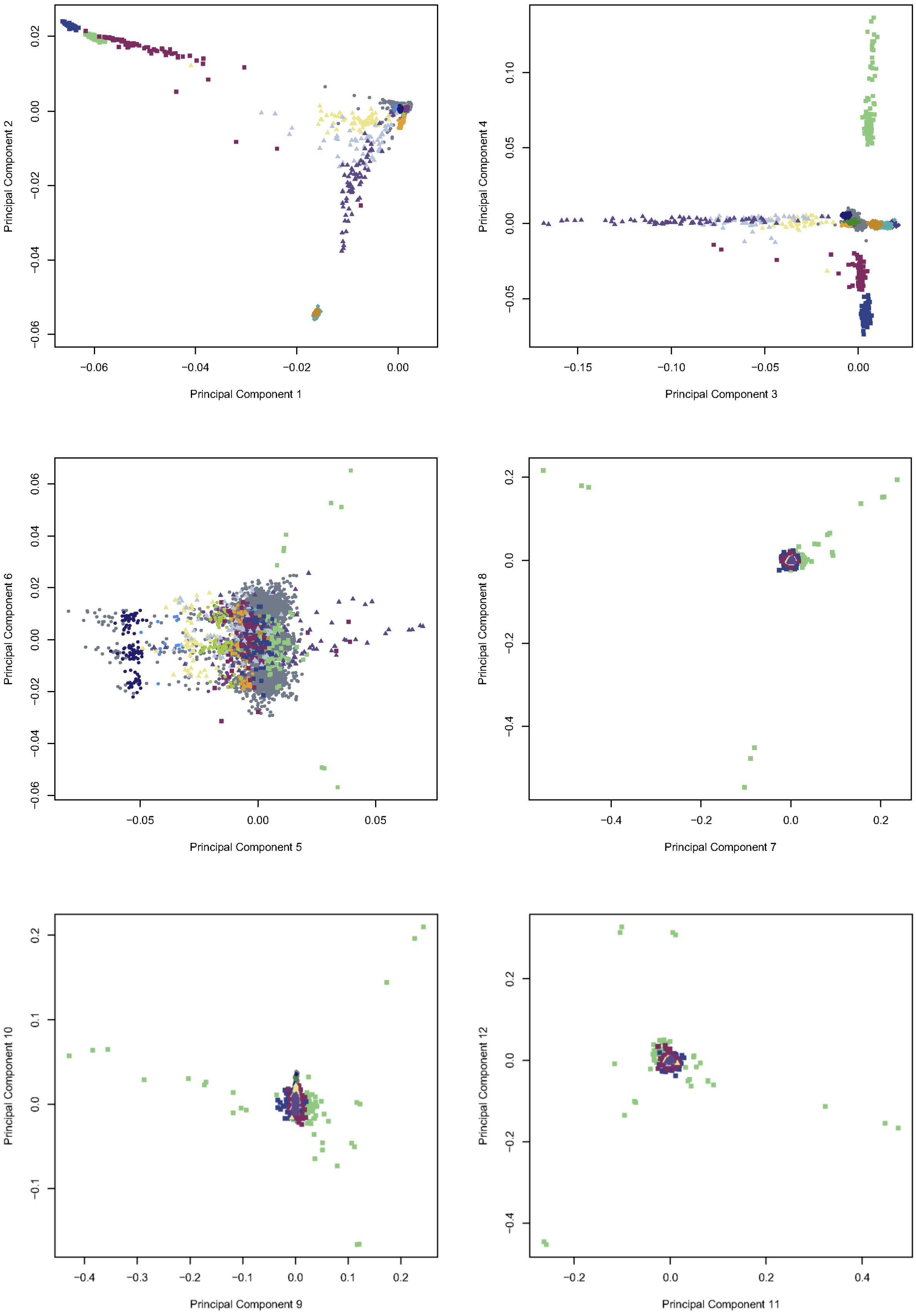
Principal Component 1
Principal Component 2
−0.06
−0.04
−0.02
0.00
0.02
Principal Component 3
Principal Component 4
−0.15
−0.10
−0.05
0.00
0.05
0.10
Principal Component 5
Principal Component 6
0.04
0.06
Principal Component 7
Principal Component 8
−0.4
−0.2
0.0
0.2
Principal Component 9
Principal Component 10
−0.3
−0.1
0.1
Principal Component 11
Principal Component 12
0.4

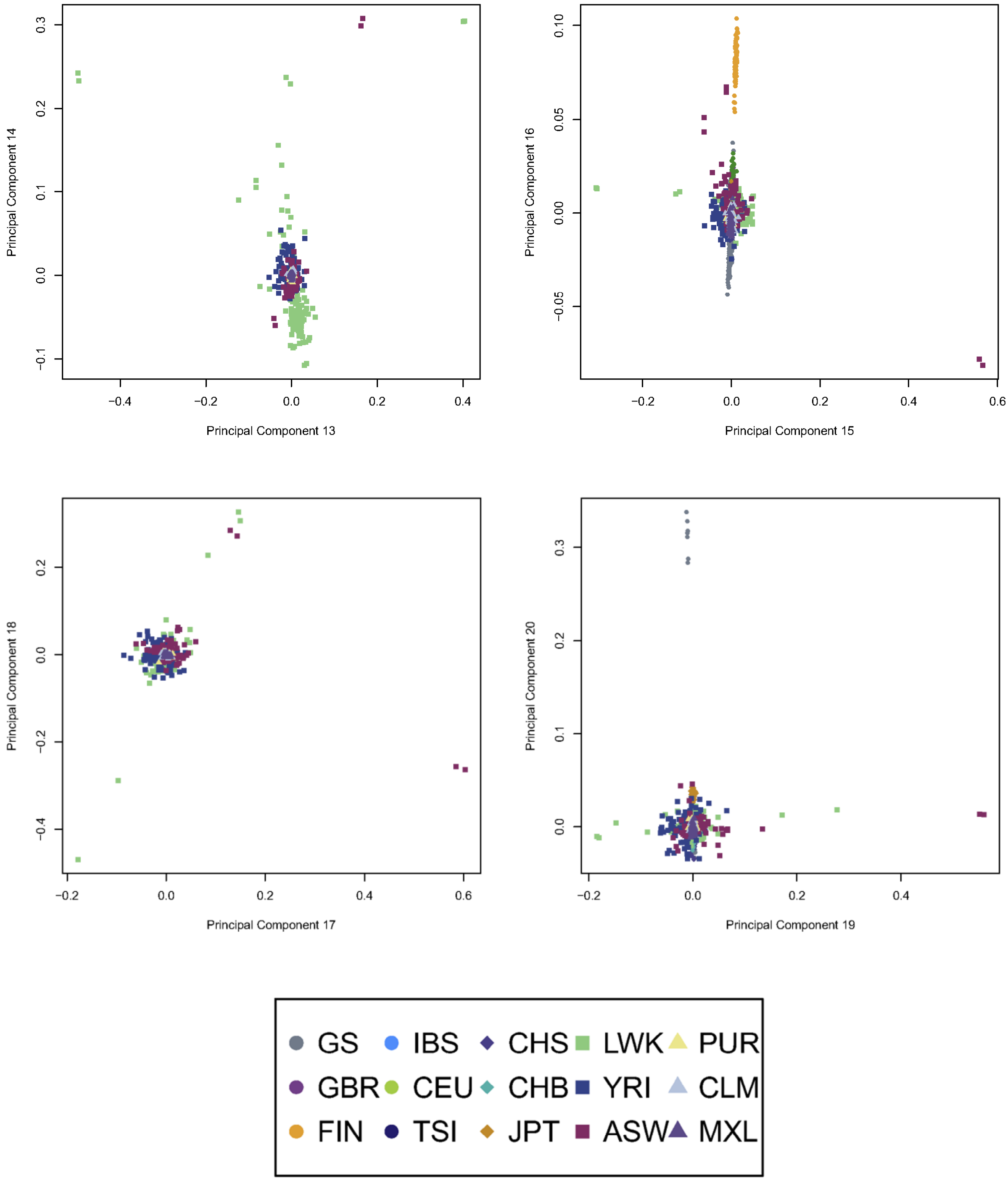

Fig. S1. Results of the PCA in the GS+1kG data set. Values for PC1 to PC20 in GS+1kG individuals.

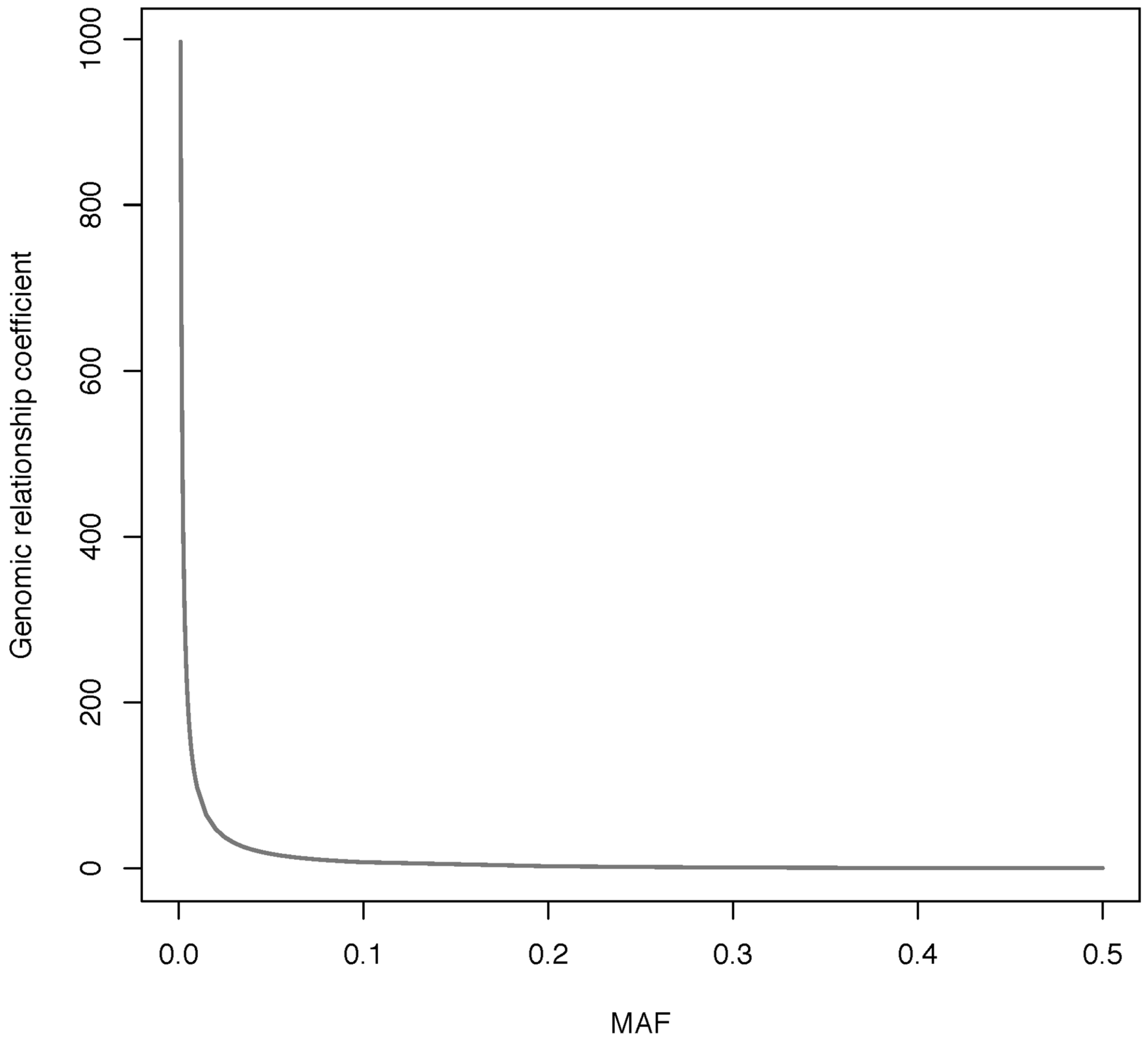

Fig S2. Expected values of pair marker scores between pairs of heterozygous individuals obtained according to the formula in Yang et al. (2010) for different values of minor allele frequencies (MAF).

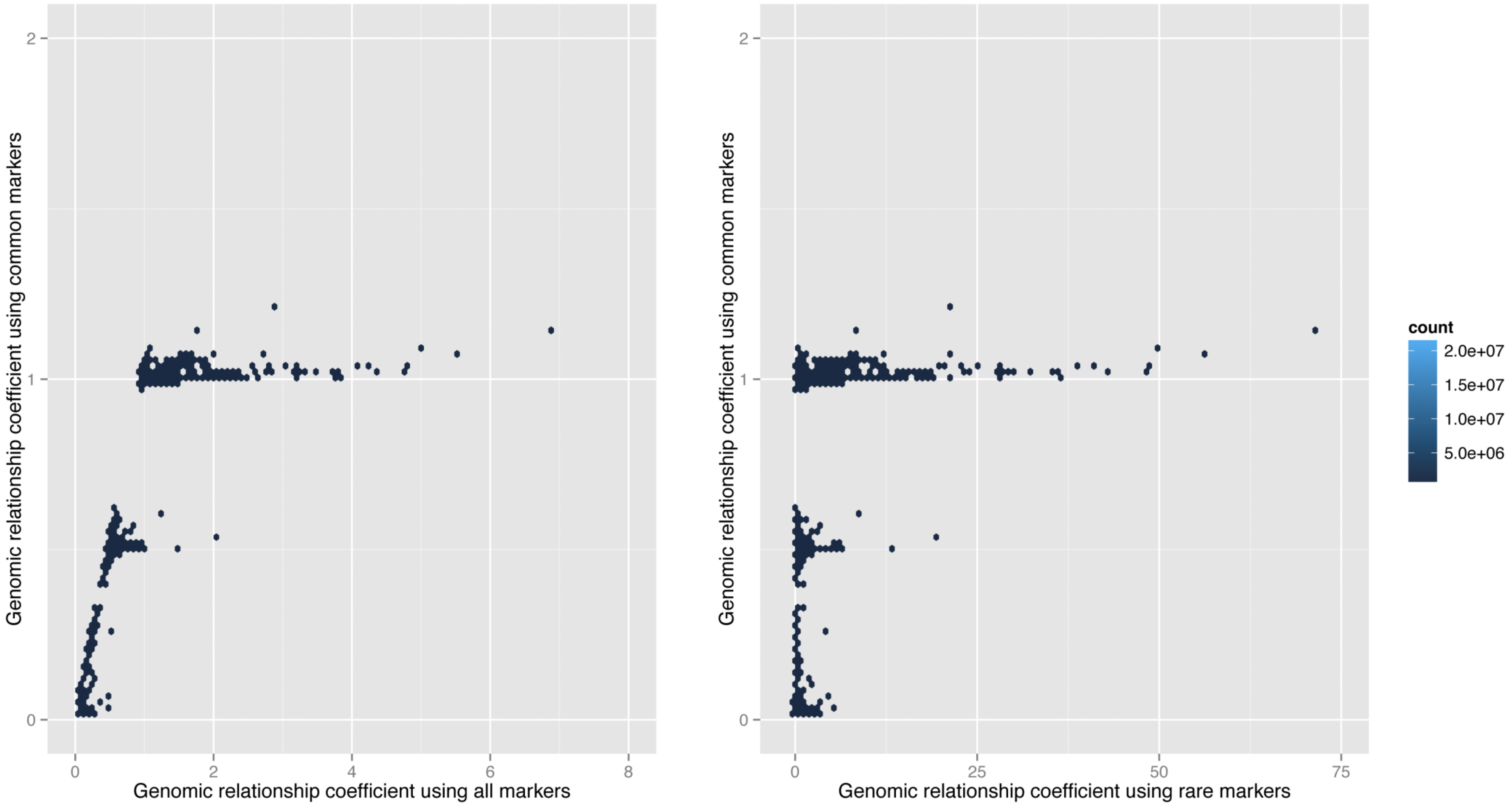

Figure S3. Comparison between values of the genomic relationship coefficients when using common markers vs using all the markers (left) or only rare markers (right).

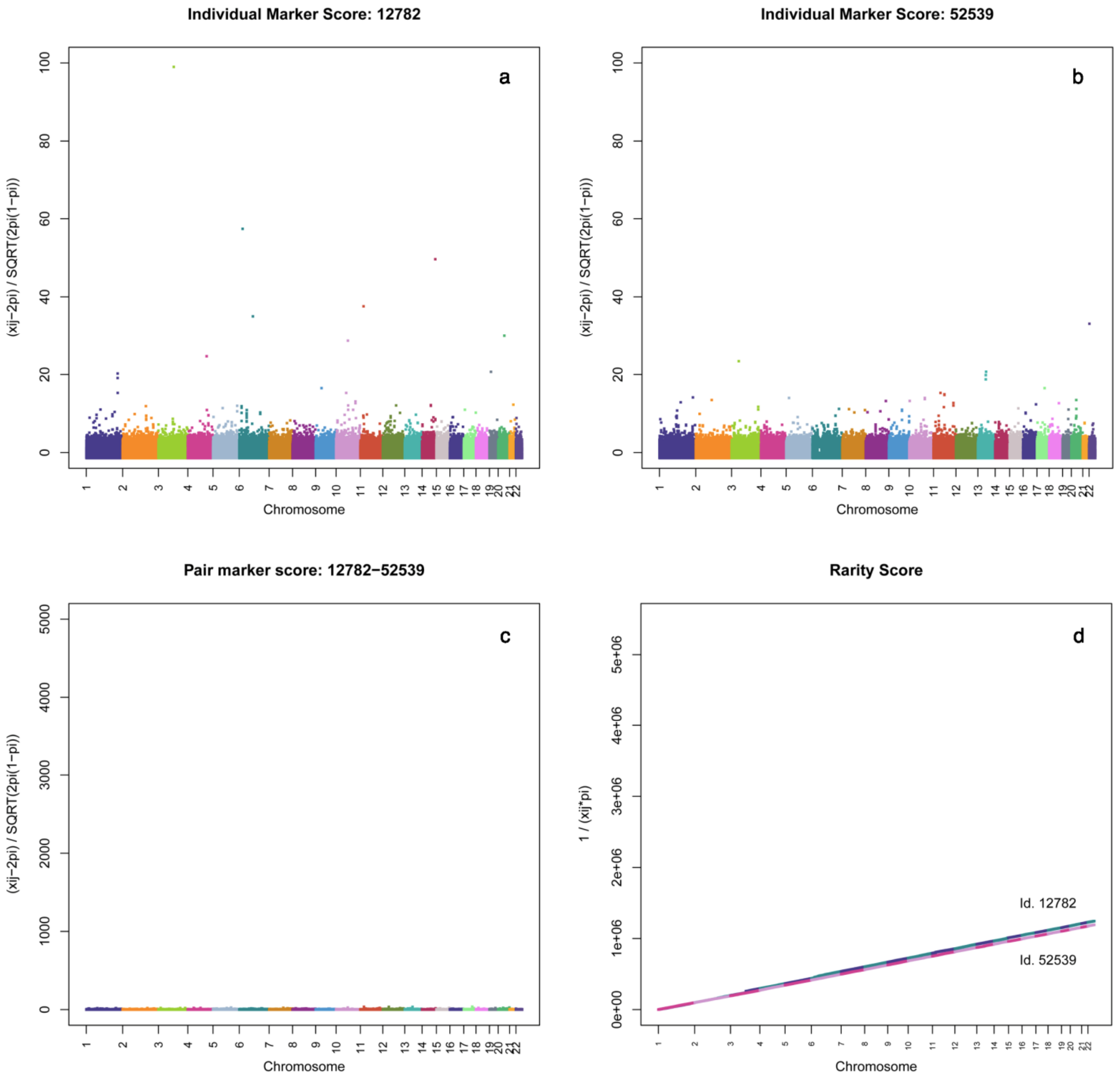

Figure S4. *Score* values of individuals 12782 and 52539. a) Individual marker score of individual 12782; b) Individual marker score of individual 52539; c) Pair marker score of individuals 12782 and 52539; d) Rarity scores of individual 12782 and 52539.

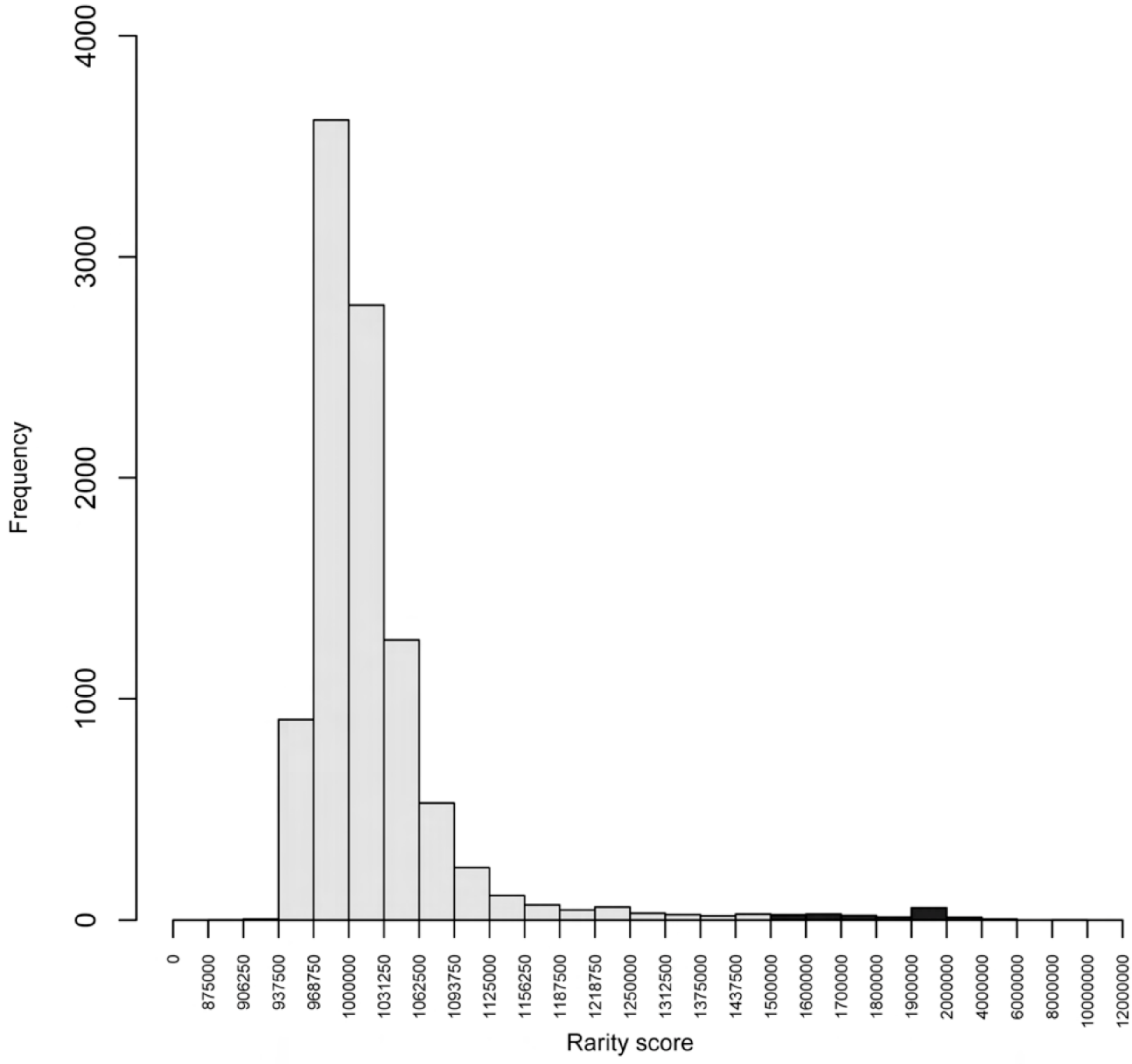

Figure S5. *Rarity score* distribution in GS:SFHS. Histogram for the *rarity score* values in all the individuals of GS:SFHS (note the change in the *total score* scale after a value of 1500000, darker bars).

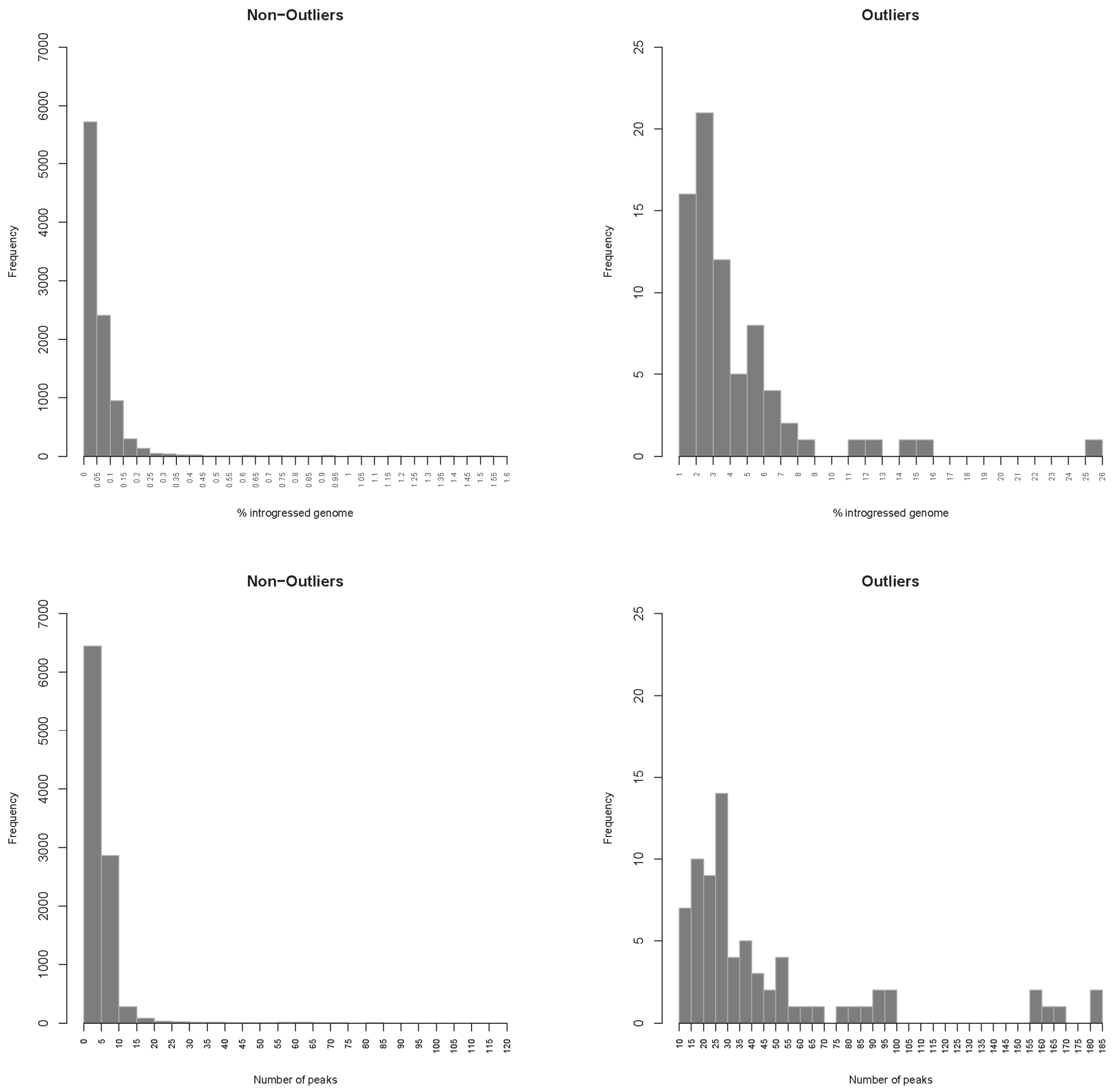

Figure S6. Distribution of the percentage of introgressed genome and number of peaks. Histograms showing the distributions of the percentage of introgressed genome and the number of peaks in GS:SFHS individuals of the two groups (left: regular individuals, right: outliers).

Figure S7

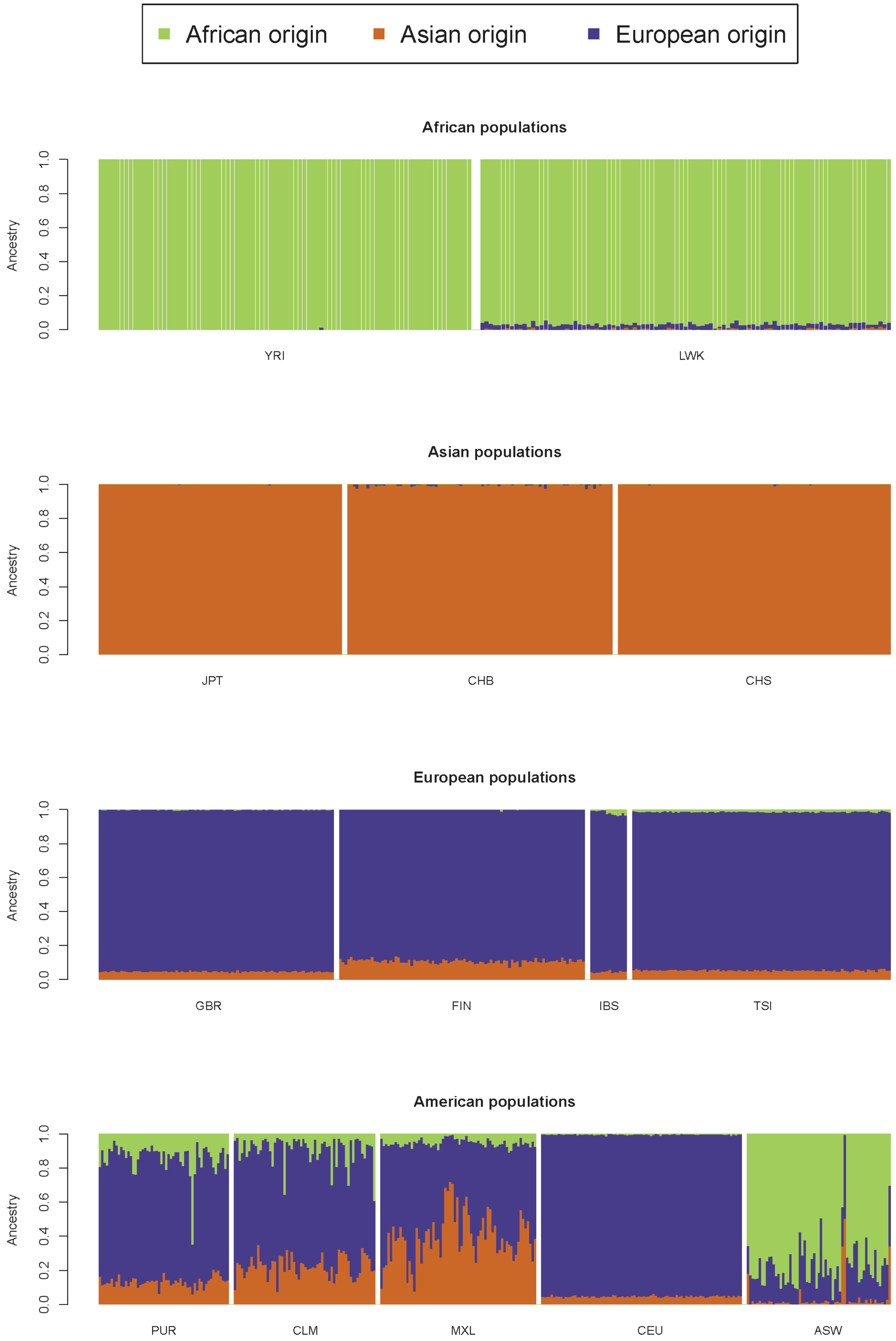

African origin
Asian origin
European origin
African populations
Ancestry
0.0
0.2
0.4
0.6
0.8
1.0
YRI
LWK
Asian populations
Ancestry
JPT
CHB
CHS
European populations
Ancestry
GBR
FIN
IBS
TSI
American populations
Ancestry
PUR
CLM
MXL
CEU
ASW

Figure S7

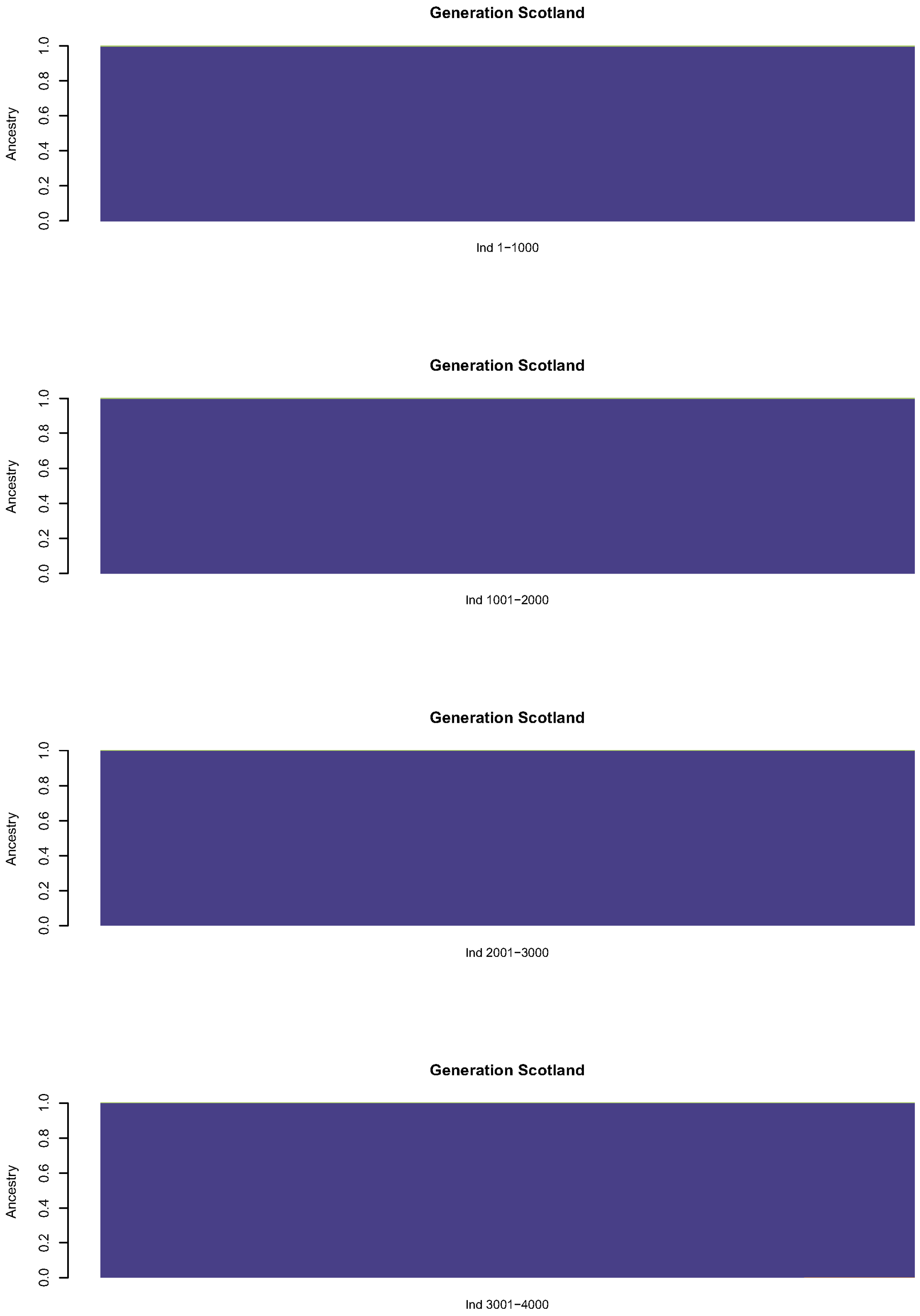
Generation Scotland
Ancestry
0.0 0.2 0.4 0.6 0.8 1.0
Ind 1−1000
Generation Scotland
Ancestry
0.0 0.2 0.4 0.6 0.8 1.0
Ind 1001−2000
Generation Scotland
Ancestry
0.0 0.2 0.4 0.6 0.8 1.0
Ind 2001−3000
Generation Scotland
Ancestry
0.0 0.2 0.4 0.6 0.8 1.0
Ind 3001−4000

Figure S7

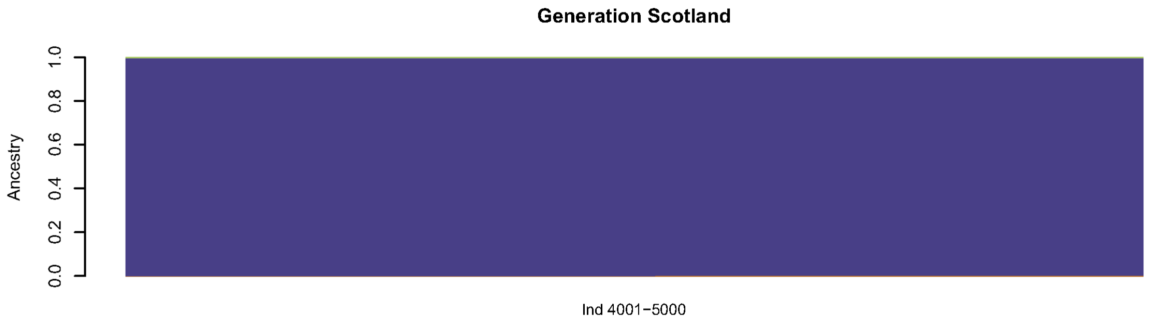
Generation Scotland
Ancestry
0.0
0.2
0.4
0.6
0.8
1.0
Ind 4001−5000

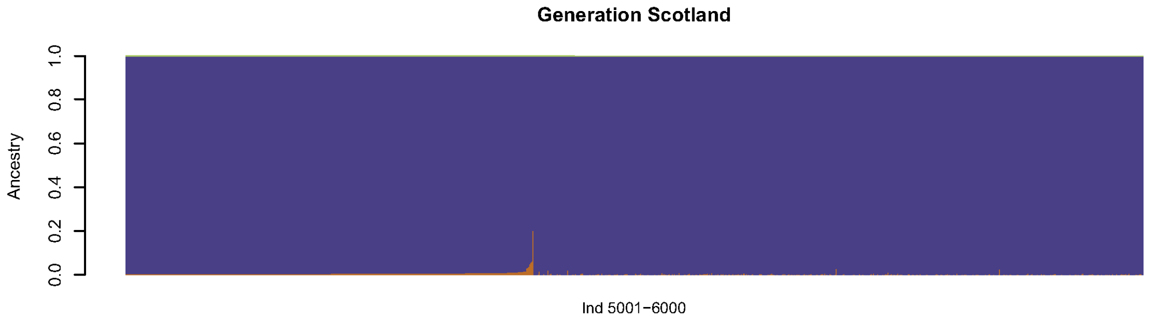
Generation Scotland
Ancestry
0.0
0.2
0.4
0.6
0.8
1.0
Ind 5001−6000

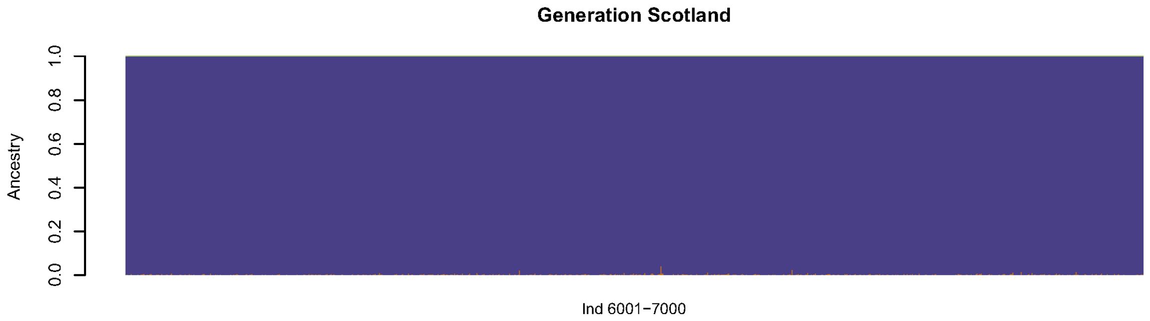
Generation Scotland
Ancestry
0.0
0.2
0.4
0.6
0.8
1.0
Ind 6001−7000

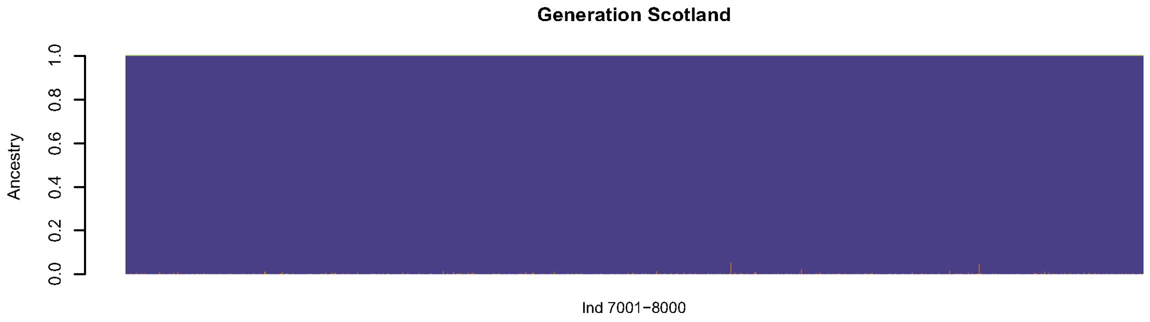
Generation Scotland
Ancestry
0.0
0.2
0.4
0.6
0.8
1.0
Ind 7001−8000

Figure S7

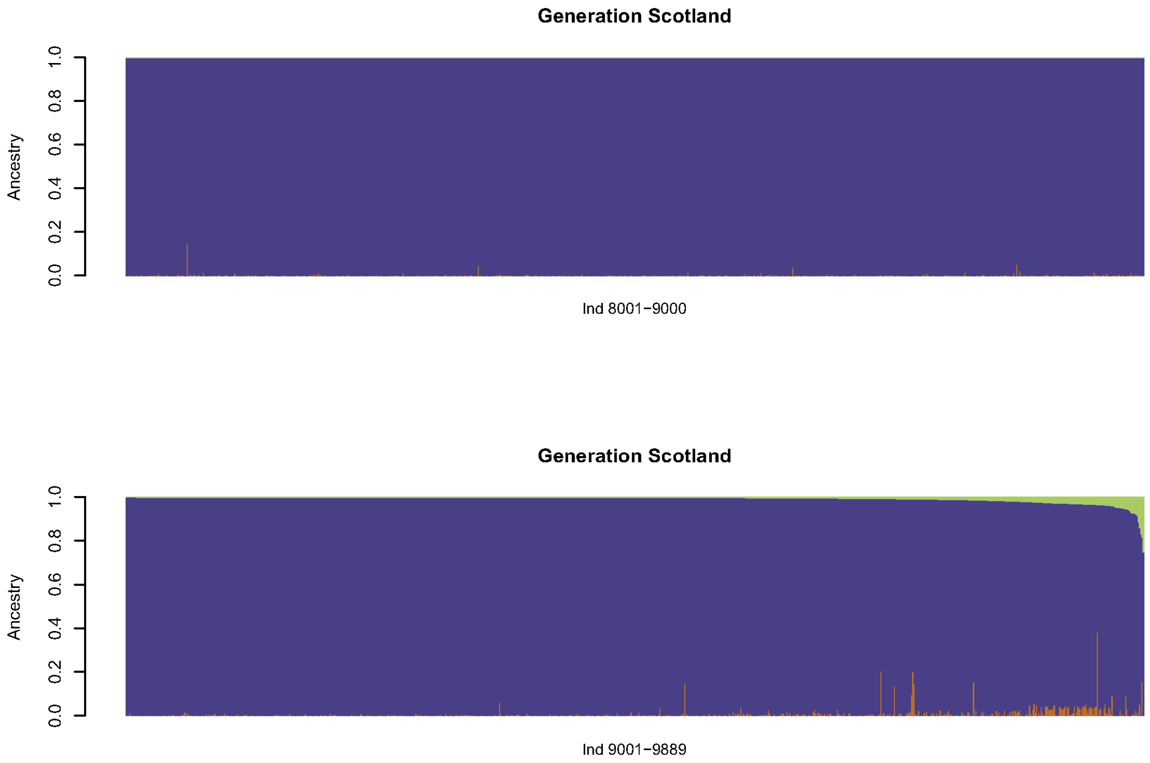

Figure S7. Proportion of each of the three ancestry origins in individuals from the GS+1kG data set from the ADMIXTURE analysis. Individuals in GS:SFHS are ordered by percentage of non-European ancestry estimated.

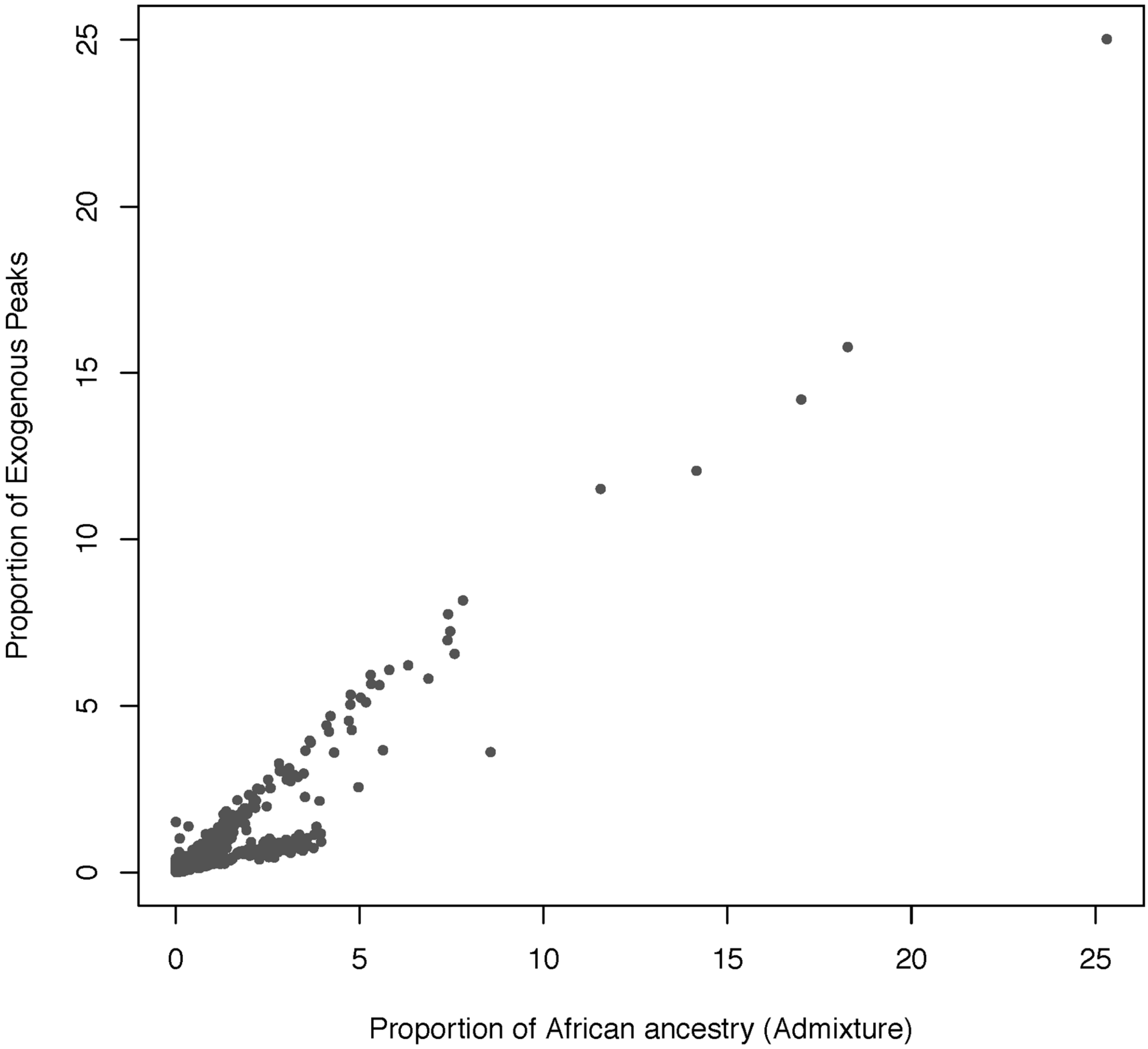

Figure S8. Correlation between the proportion of exogenous peaks and the proportion of African ancestry estimated using ADMIXTURE**.**

Figure S9

Chromosome 1

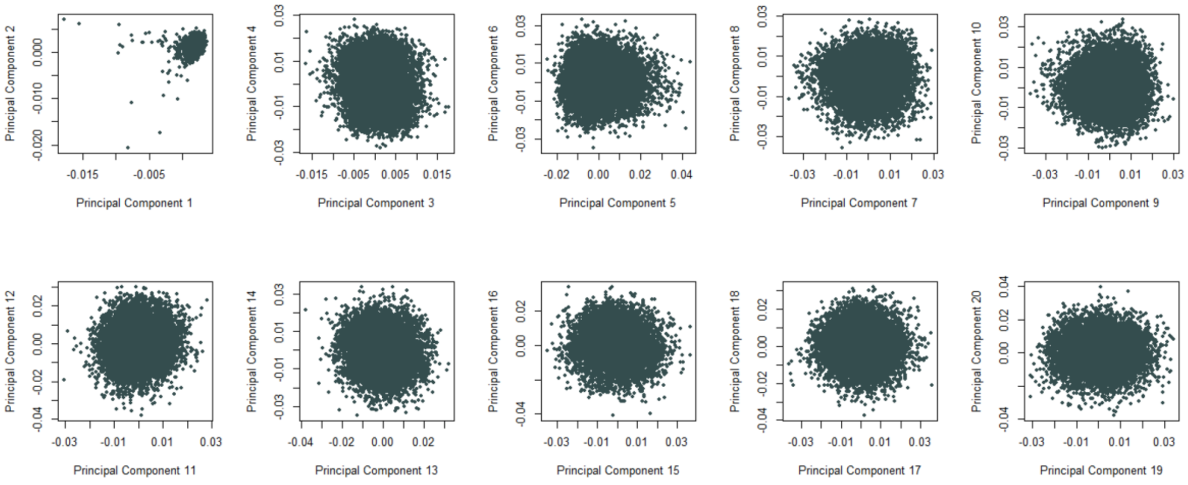

Chromosome 2

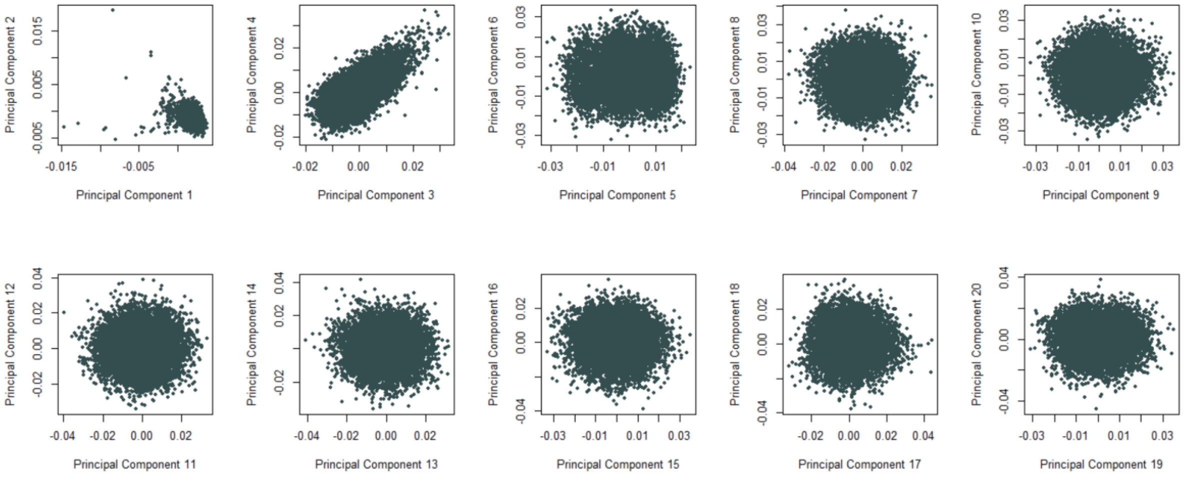

Chromosome 3

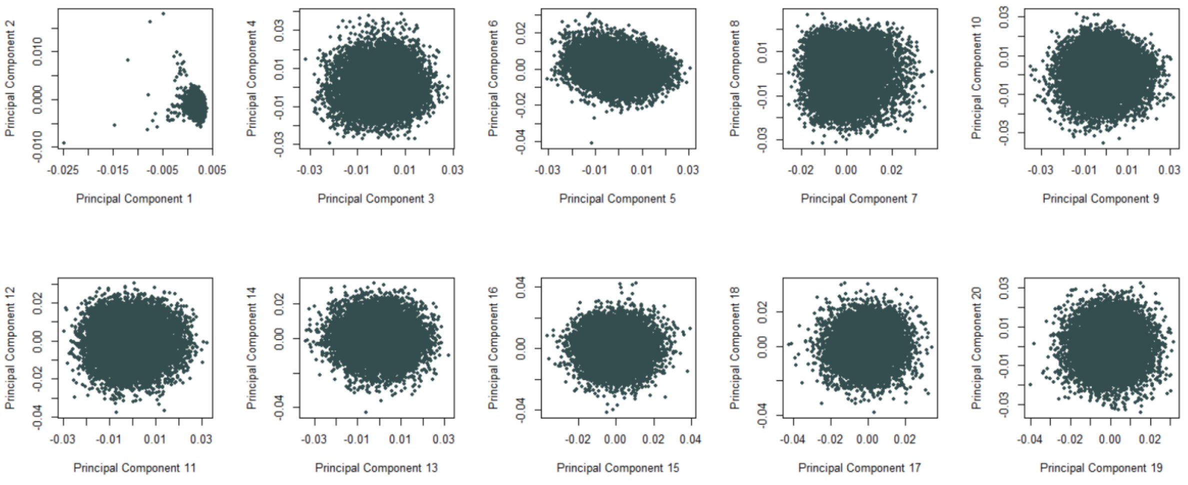

Figure S9

Chromosome 4

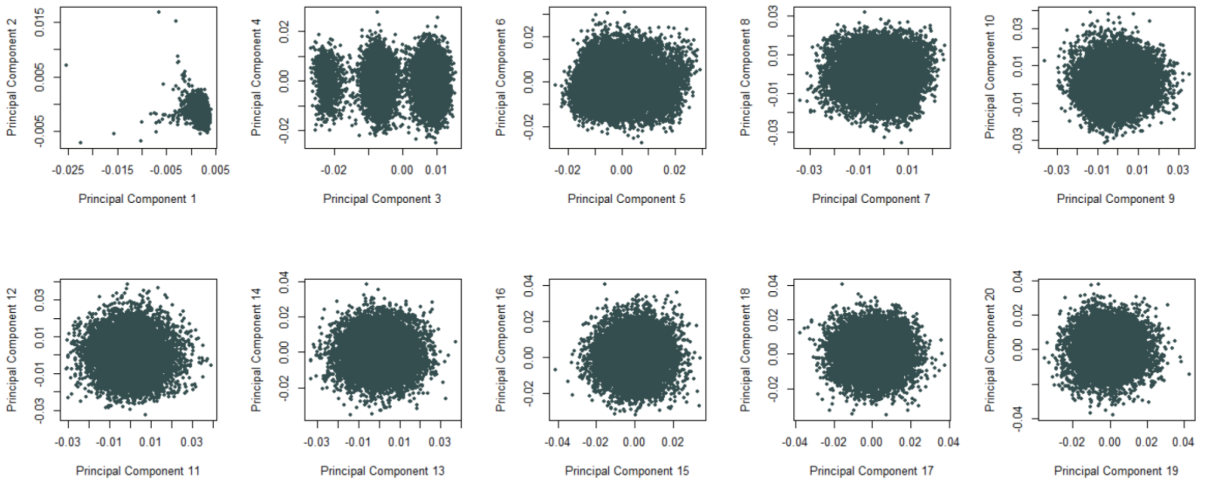

Chromosome 5

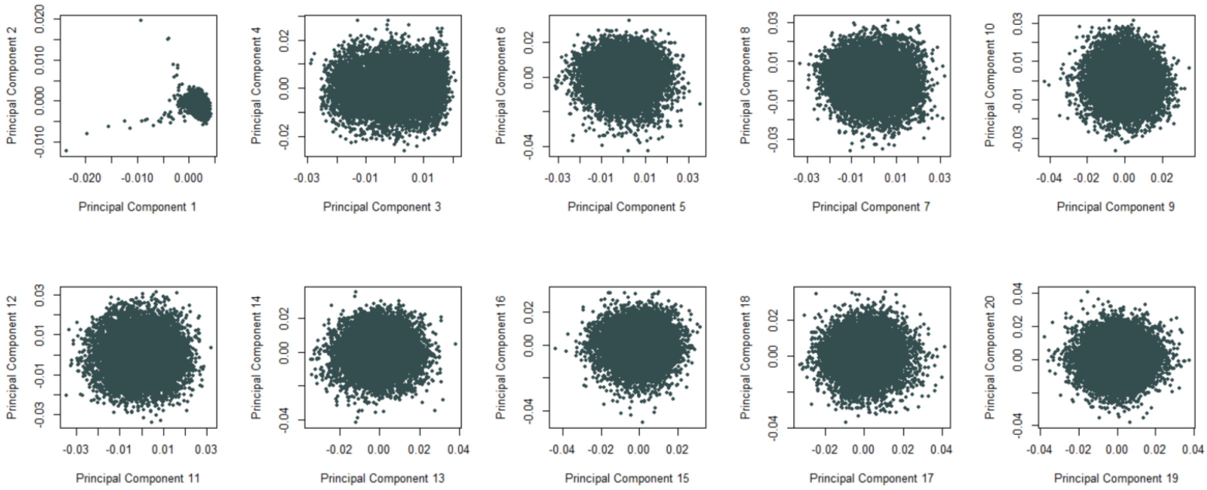

Chromosome 6

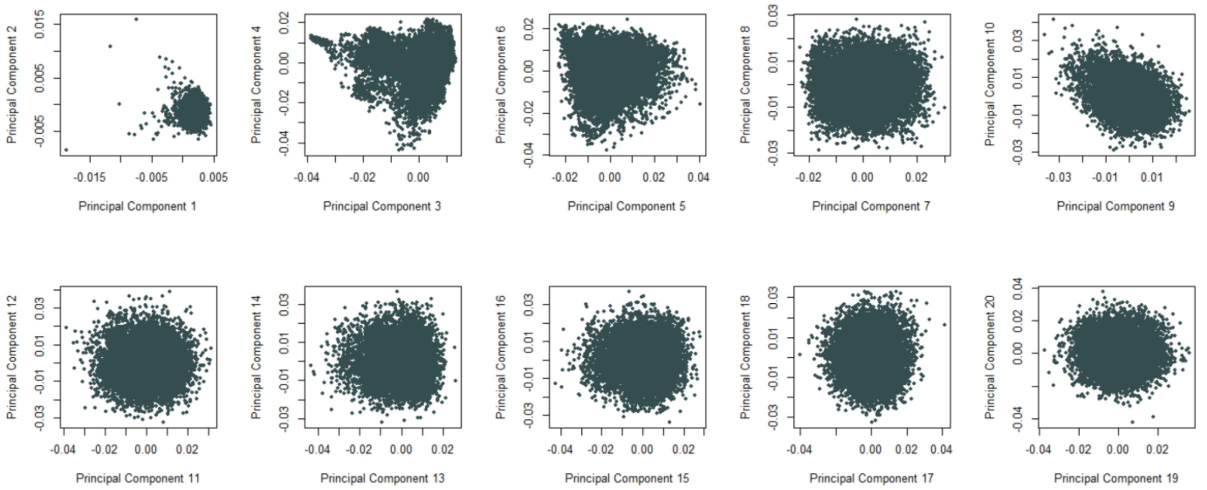

Figure S9

Chromosome 7

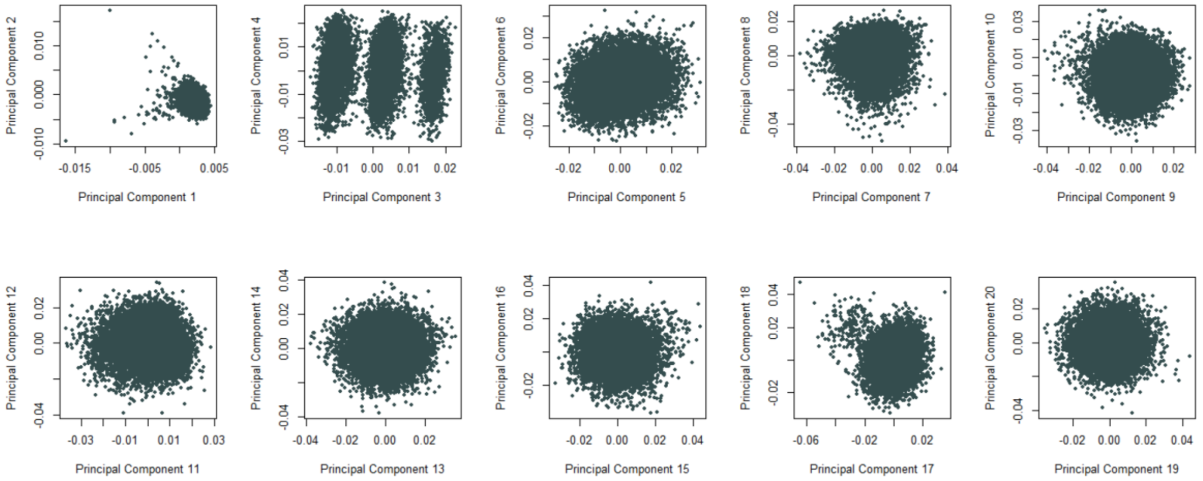

Chromosome 8

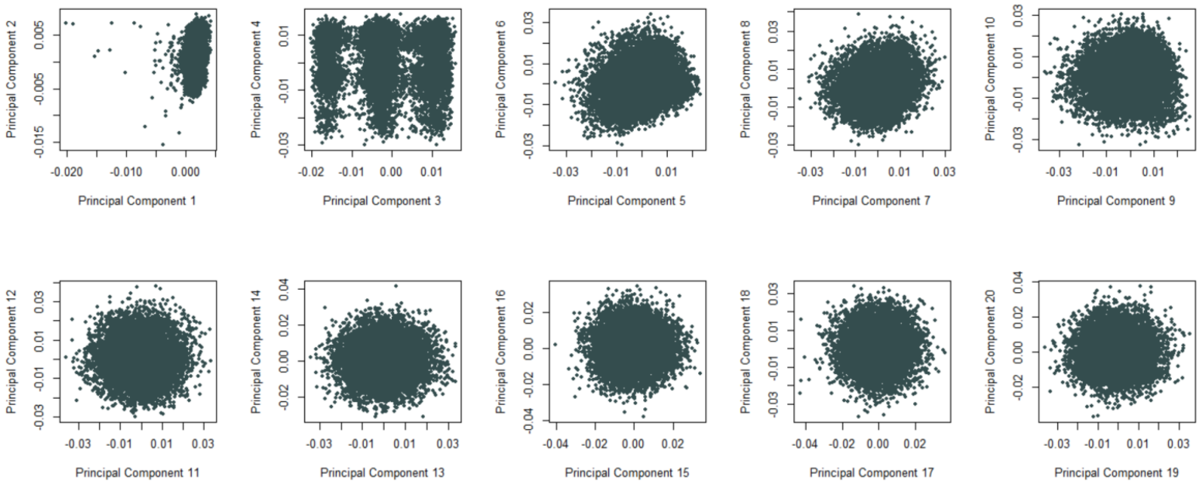

Chromosome 9

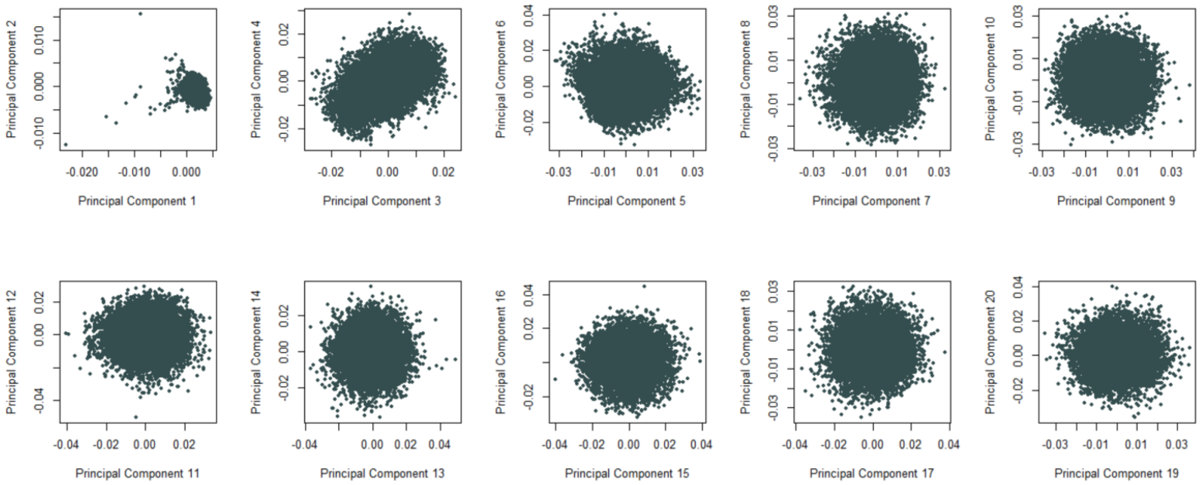

Figure S9

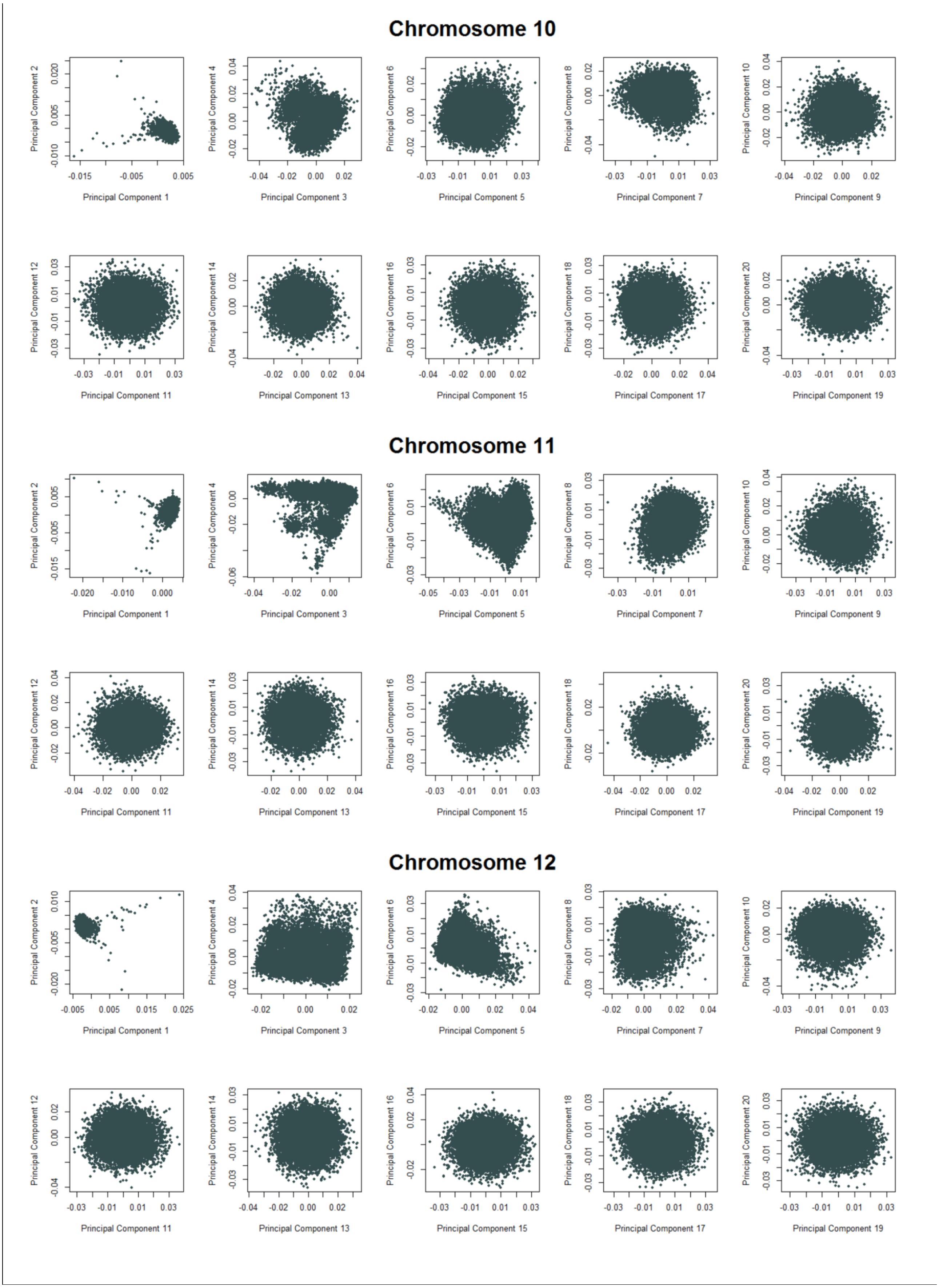
Chromosome 10
Principal Component 1
Principal Component 2
Principal Component 3
Principal Component 4
Principal Component 5
Principal Component 6
Principal Component 7
Principal Component 8
Principal Component 9
Principal Component 10
Principal Component 11
Principal Component 12
Principal Component 13
Principal Component 14
Principal Component 15
Principal Component 16
Principal Component 17
Principal Component 18
Principal Component 19
Principal Component 20
Chromosome 11
Chromosome 12

Figure S9

Chromosome 13

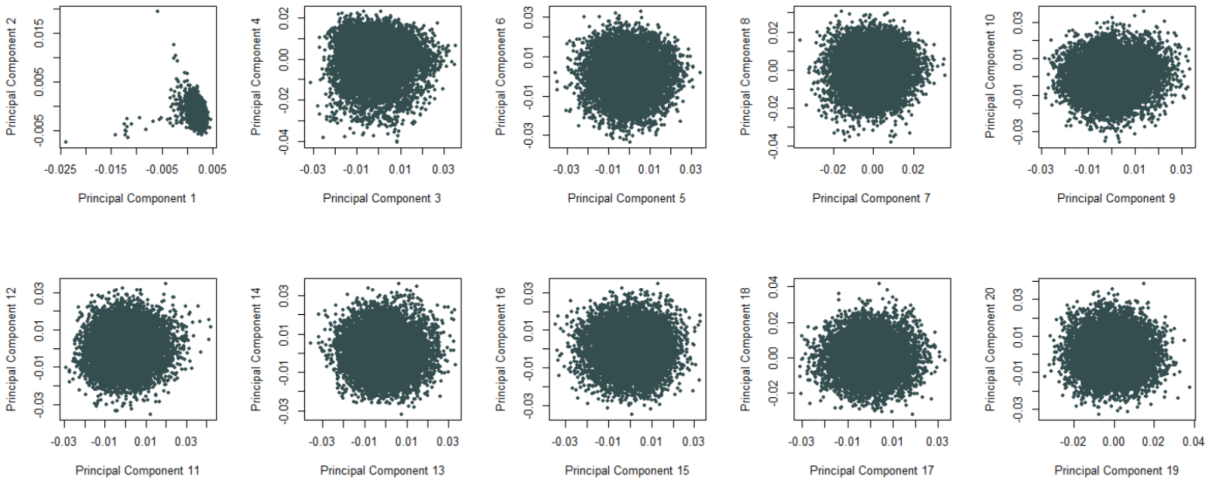

Chromosome 14

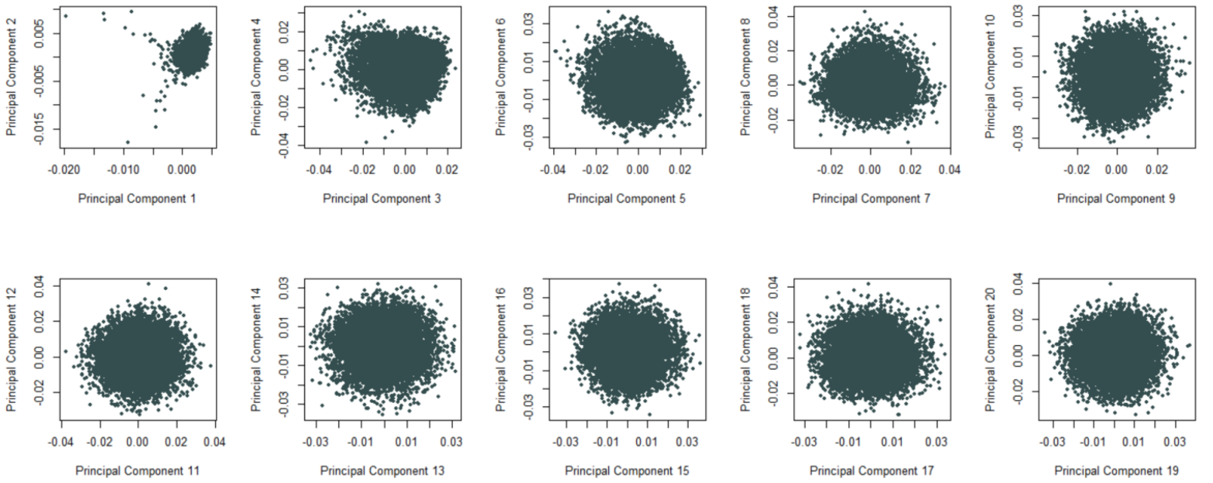

Chromosome 15

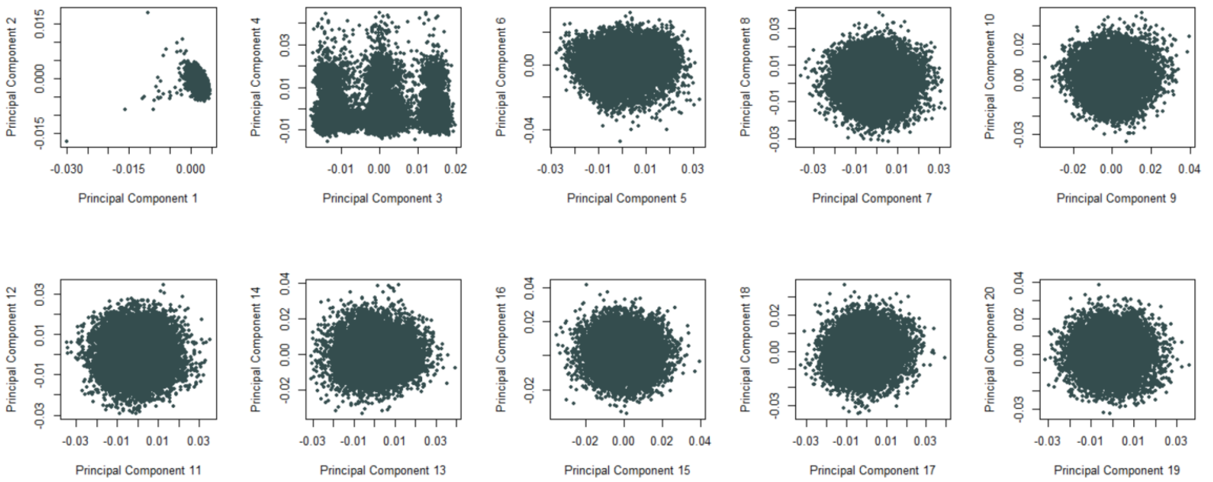

Figure S9

Chromosome 16

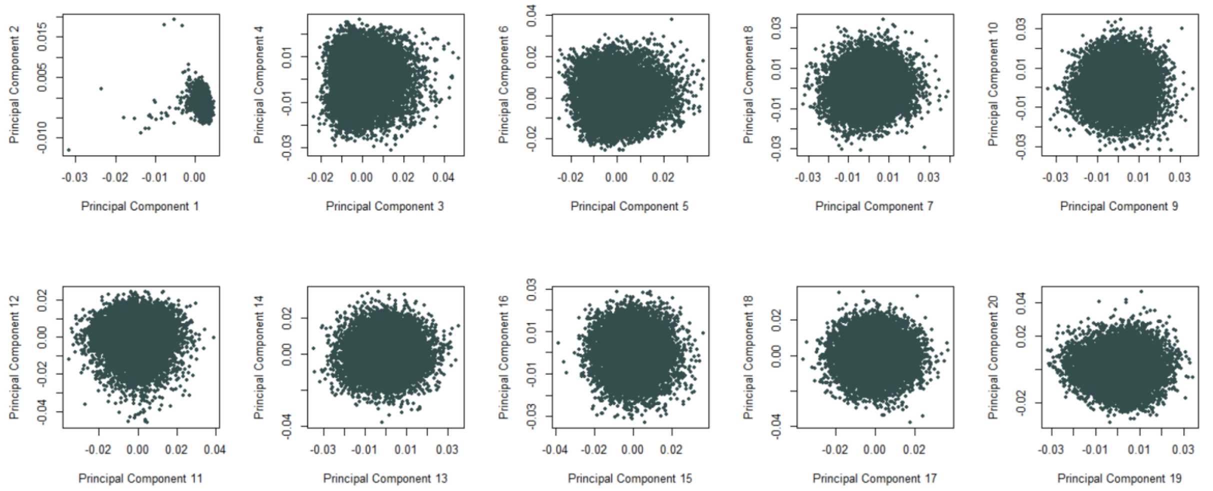

Chromosome 17

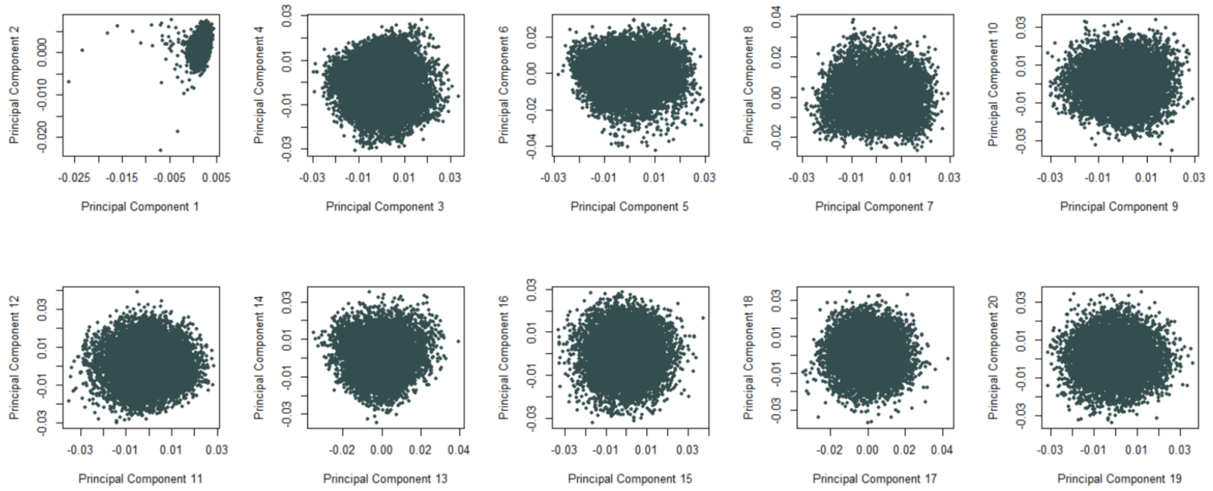

Chromosome 18

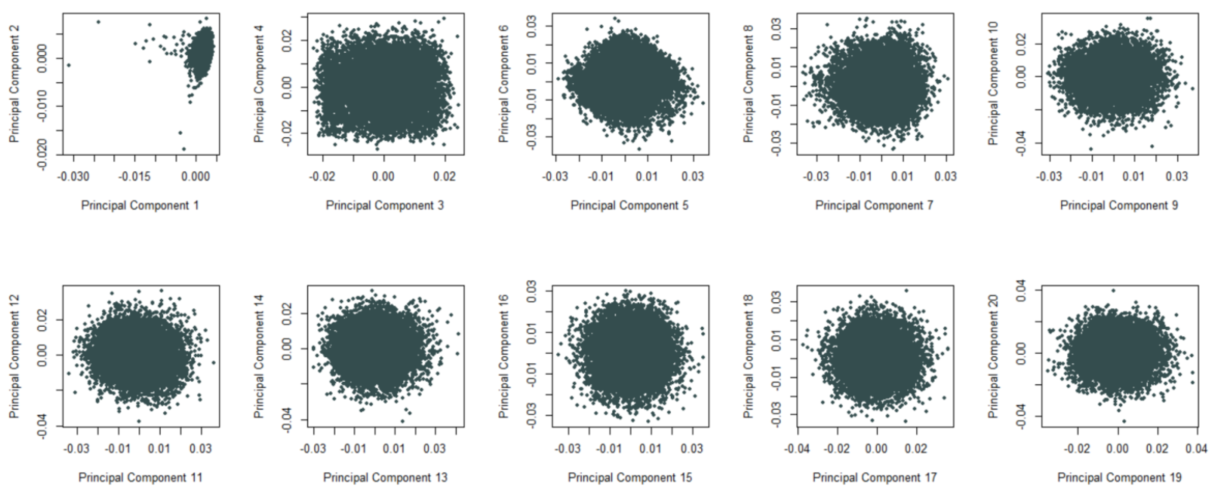

Figure S9

Chromosome 19

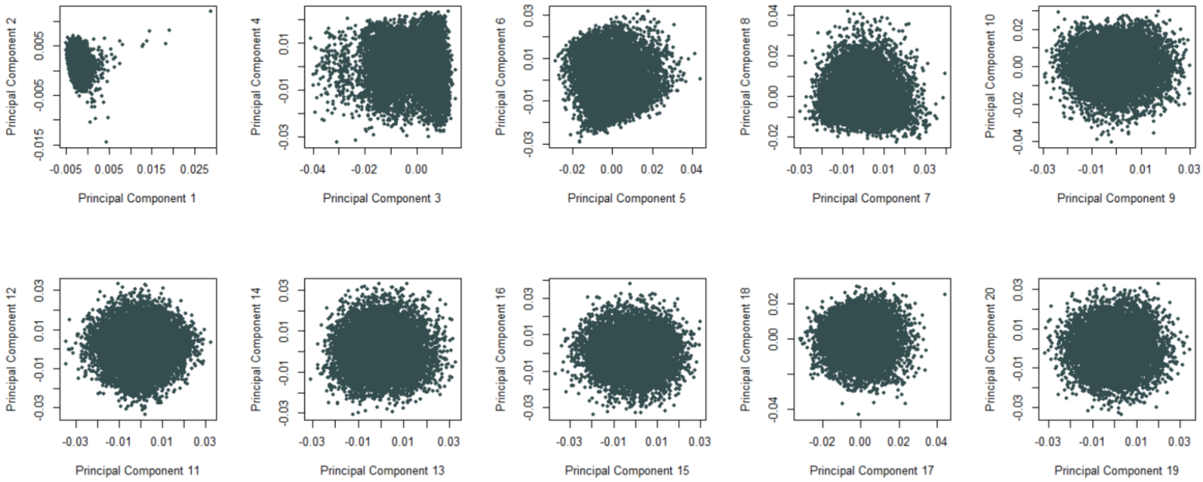

Chromosome 20

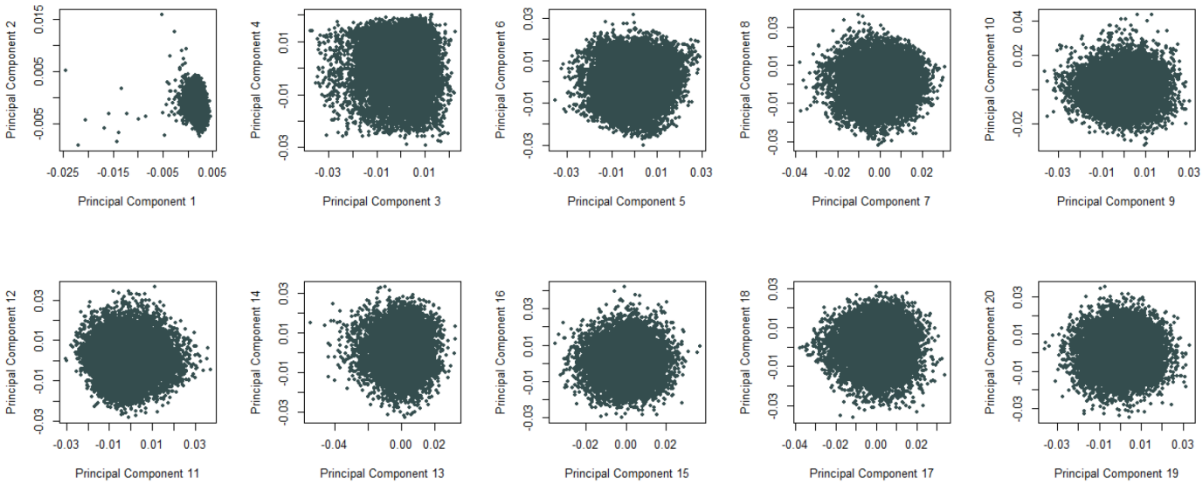

Chromosome 21

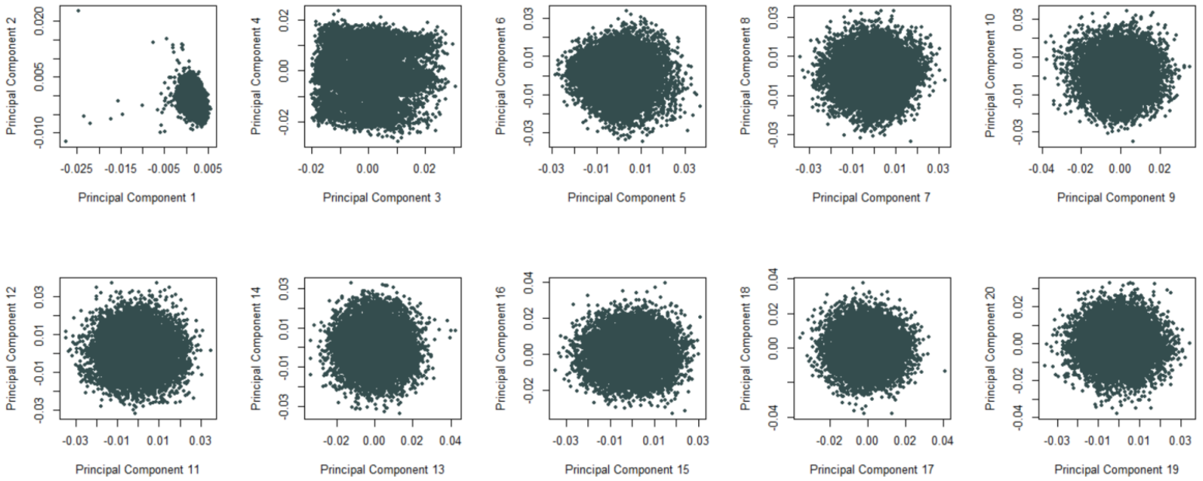

Figure S9

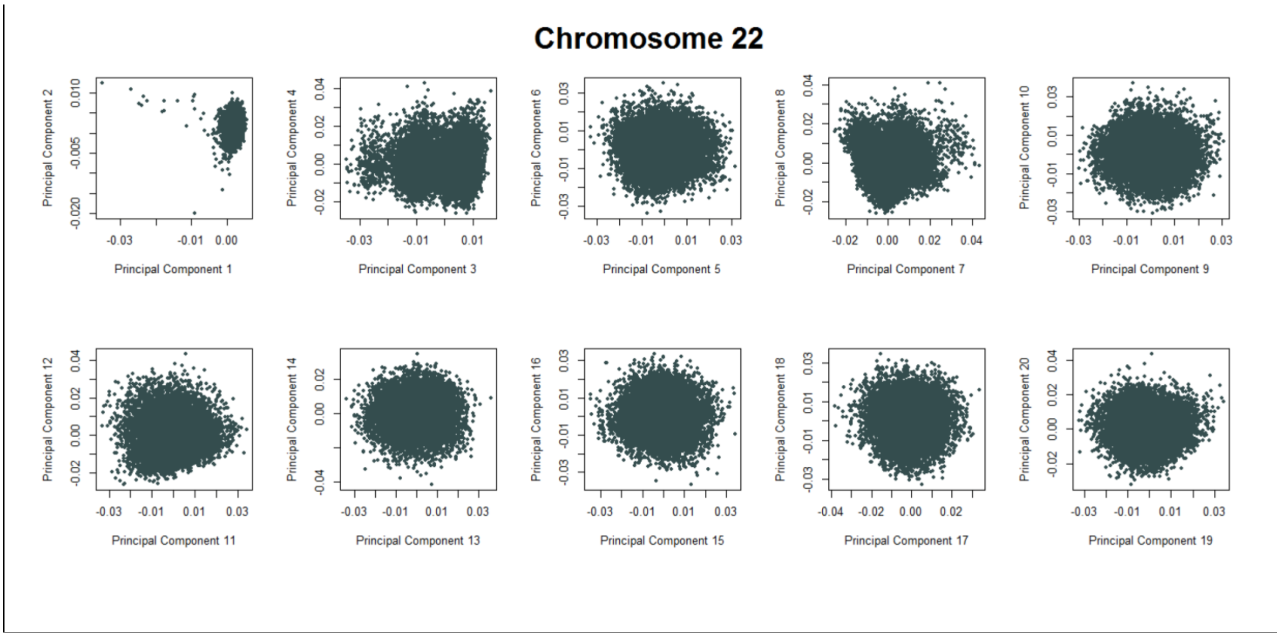

Figure S9. Results of the chromosomal PCA. Ten plots per chromosome show the values obtained for PC 1 to 20 per each of the 22 autosomes

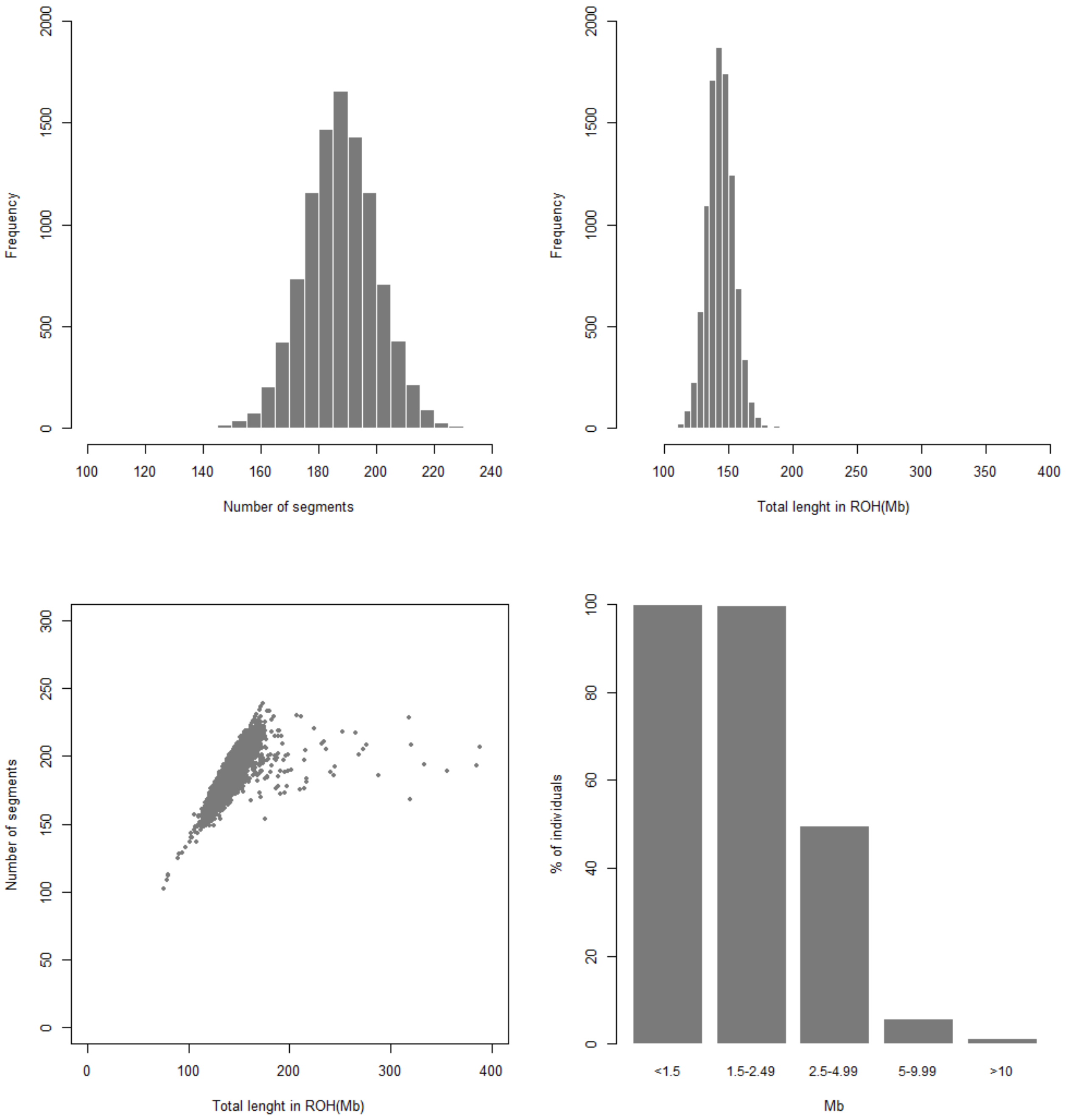

Figure S10. Summary of the results of the ROH analysis in GS:SFHS. a) distribution of the number of segments per individual in the cohort; b) distribution of the total length of homozygous segments per individual; c) number of ROH compared to the total length of ROH per individual; d) proportion of individuals with one or more ROH of a given length.

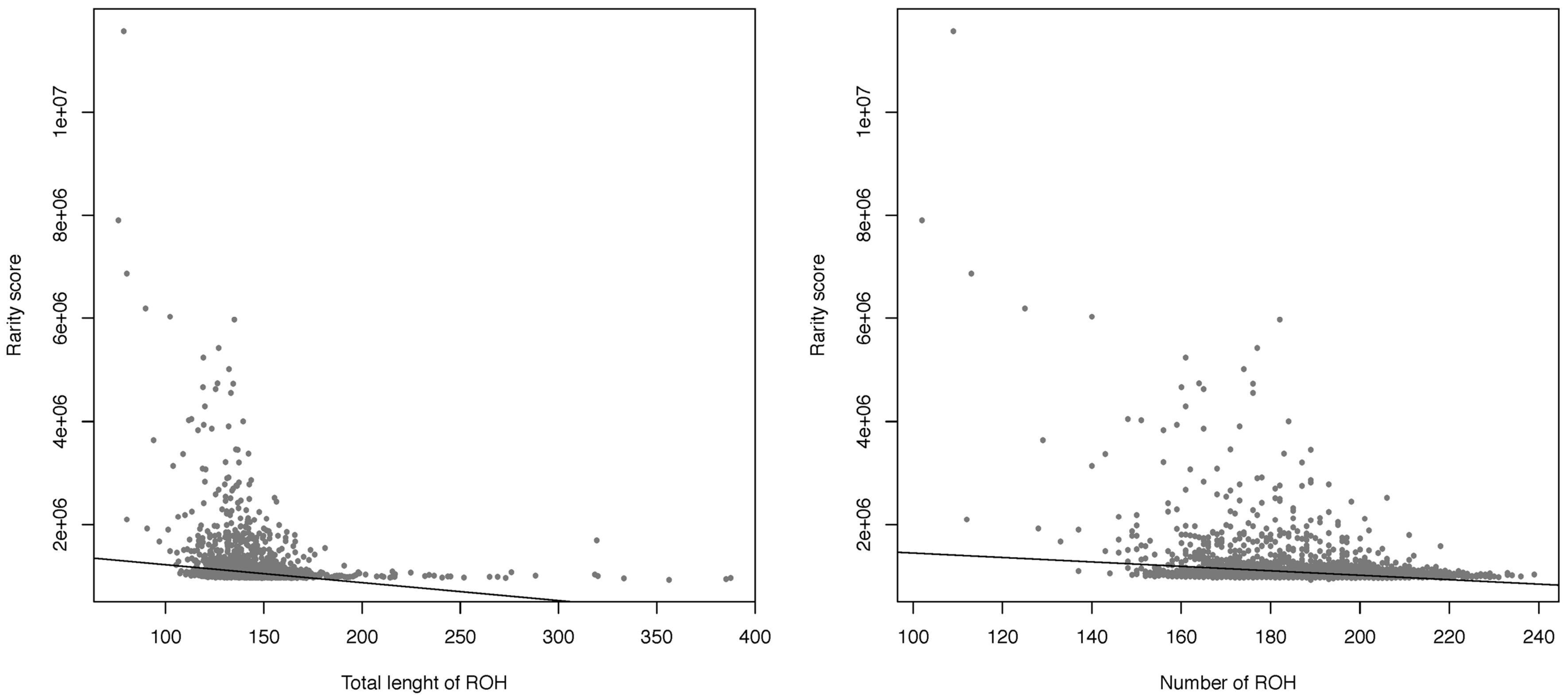

Figure S11. Relationship between rarity scores and ROH. Correlation between the values for the rarity score of GS:SFHS individuals and their total length of ROH (left) or their number of ROH (right).

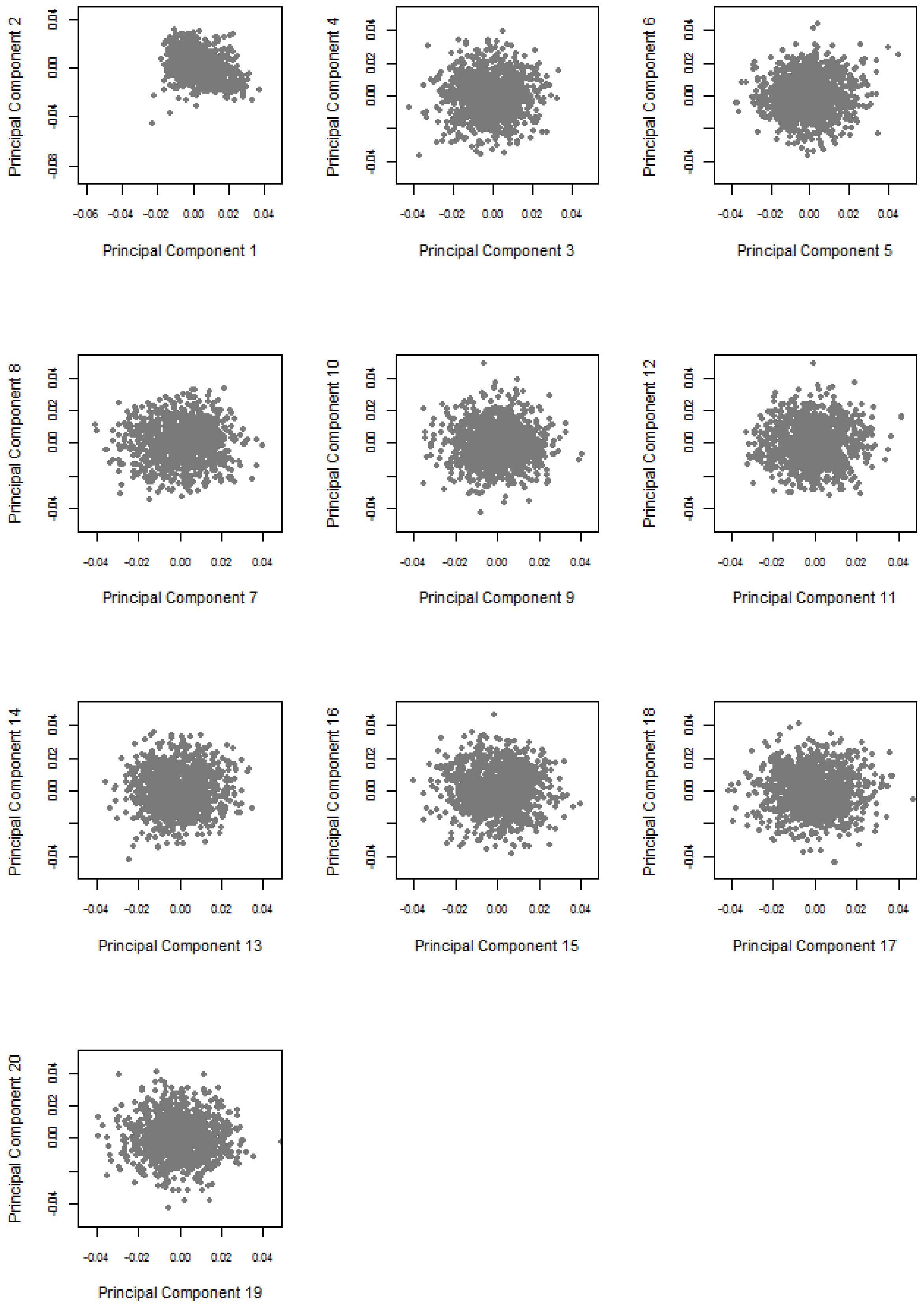

Figure S12. Results of the PCA in the GS:SFHS data set (After removing familiar structure, individuals with mixed backgrounds and Italian ancestry) Values for PC1 to PC20 in GS:SFHS individuals.

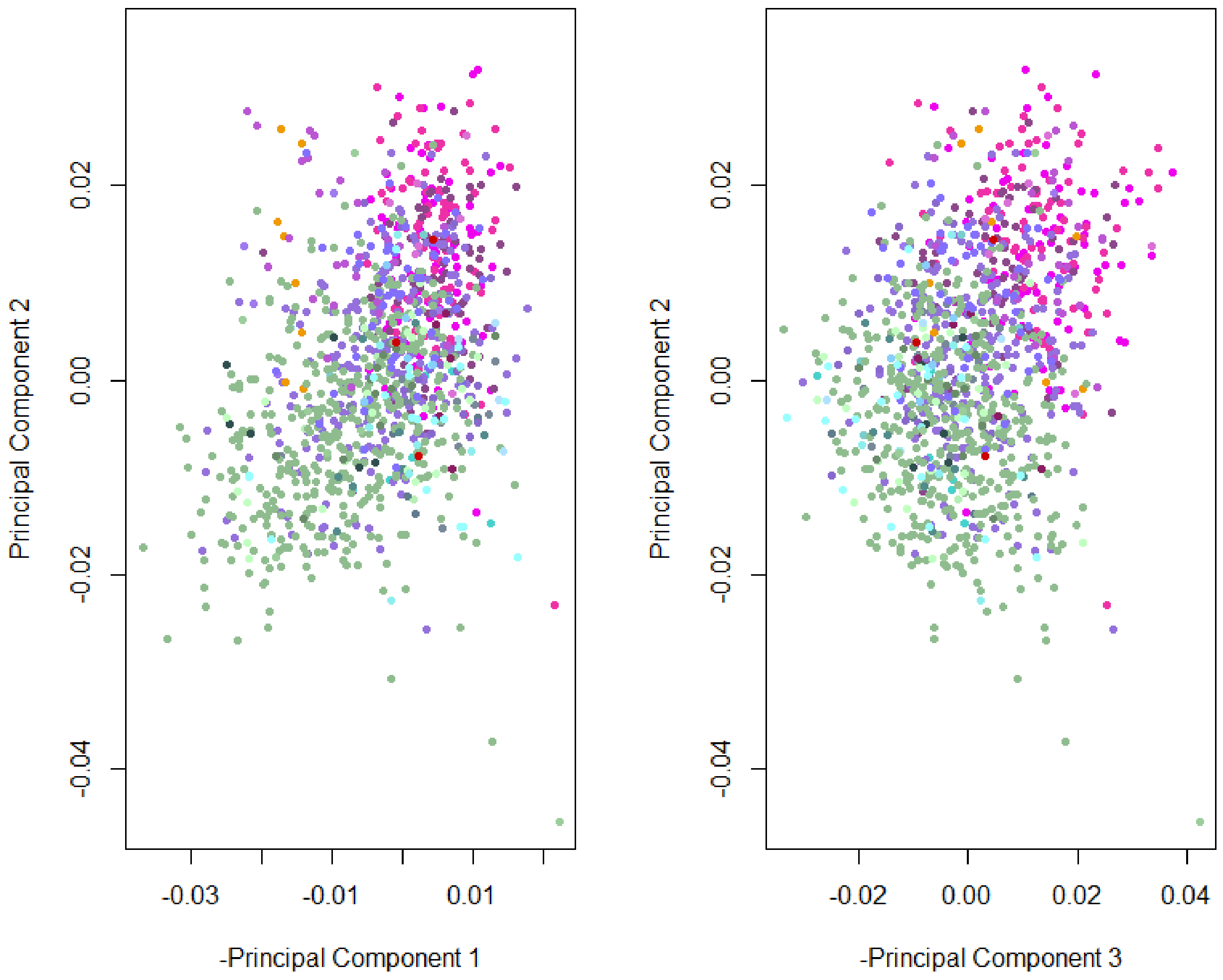

Figure S13. Results of the PCA in the GS:SFHS grandparents subset. Values for –PC1 and PC2 (left) and -PC3 and PC2 (right) of the 1113 individuals with 4 grandparents from the same origin. The colours of the points correspond to the origin of the grandparents of the individuals corresponding to coding in Fig. 7a.

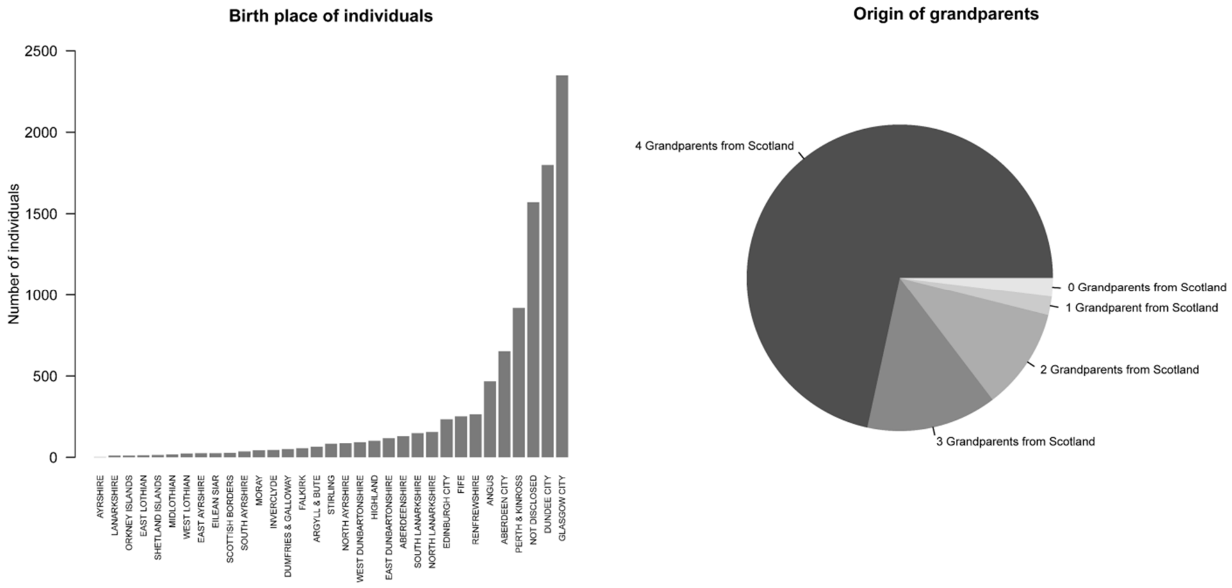

Figure S14. City of birth of the 9889 individuals of the GS:SFHS data set (right) and number of grandparents from other regions than Scotland (for those individuals with information about the 4 grandparents N=7725) (left).

SUPPLEMENTAL TABLES

Table S1. Results for regions responsible of the PCs forming three-cluster patterns detected in the GWAS analysis: Chromosome, number of the PC showing the pattern, length of the area (in base pairs), chromosomal region where it is located and genes located in them (including complete name and known functions).

-see file SuppTableS1.xlsx-

Table S2. Thresholds and number of markers included in the computation of the GRMs in GS+1kG population.

**Number of markers**				
**All markers**	**MAF>1%**	**MAF>5%**	**MAF<1%**	**MAF<5%**
635,190	595,295	550,611	39,895	84,579

Table S3. Proportion of the phenotypic variance captured by genome-wide SNPs (summarised in a genomic relationship matrix, GRM) and by proximity between individuals according to predicted latitude and longitude (summarised in a similarity matrix, Geo), with (right) and without (left) including current living region as a fixed effect in four health-related traits. The P values show the significance of the log-likelihood ratio test comparing the model including only matrix GRM vs including also matrix Geo.

**Trait**	**Sex + Age + Age^2**			**Sex + Age + Age^2 + Current Region**		
	**V(GRM)**	**V(GEO)**	**Pval**	**V(GRM)**	**V(GEO)**	**Pval**
**BMI**	0.282(0.053)	0.014(0.012)	2.83E-03	0.277(0.054)	0.011(0.010)	1.02E-02
**Fat**	0.308(0.055)	0.027(0.018)	6.43E-11	0.303(0.055)	0.019(0.015)	2.64E-04
**WHR**	0.199(0.053)	0.017(0.015)	3.94E-03	0.183(0.053)	0.021(0.017)	1.77E-03
**HDL**	0.274(0.052)	0.057(0.031)	1.30E-08	0.274(0.052)	0.058(0.033)	9.47E-03

Table S4. Effects of the covariates sex, age, age^2, predicted latitude, predicted longitude and current region when fitted in a mixed linear model with a genomic relationship matrix.

Fixed Effects	BMI	Fat	WHR	HDL
**Intercept**	3.158(0.497)	0.411(19.983)	-0.584(0.224)	0.212(0.743)
**Sex**	-0.027(0.004)	11.639(0.176)	-0.115(0.002)	0.220(0.007)
**Age**	0.010(0.001)	0.437(0.047)	0.004(0.001)	0.009(0.002)
**Age^2**	0.000(0.000)	-0.003(0.000)	0.000(0.000)	0.000(0.000)
**Latitude**	-0.003(0.009)	0.118(0.344)	0.006(0.004)	-0.004(0.013)
**Longitude**	-0.012(0.007)	-0.909(0.270)	-0.011(0.003)	0.010(0.010)
**Dundee**	-0.015(0.009)	-1.169(0.364)	0.016(0.004)	0.022(0.014)
**Dumfries**	-0.059(0.049)	-2.930(1.925)	0.003(0.023)	0.006(0.074)
**Edinburgh**	-0.042(0.018)	-2.285(0.721)	-0.008(0.008)	-0.002(0.027)
**Falkirk**	-0.020(0.024)	-1.628(0.965)	0.008(0.011)	-0.010(0.036)
**Glasgow**	-0.016(0.010)	-0.875(0.380)	0.007(0.004)	-0.062(0.014)
**W. Isles**	0.052(0.078)	1.526(3.086)	0.034(0.036)	-0.157(0.115)
**Inverness**	-0.099(0.045)	-5.086(1.792)	-0.037(0.021)	0.038(0.067)
**Kilmarnock**	-0.028(0.021)	-0.340(0.851)	0.015(0.010)	0.030(0.032)
**Kirkwall**	0.040(0.122)	-0.965(4.830)	0.019(0.057)	0.024(0.180)
**Kirkcaldy**	-0.028(0.017)	-1.664(0.672)	0.008(0.008)	0.039(0.025)
**Motherwell**	-0.011(0.023)	-0.797(0.936)	0.006(0.011)	-0.035(0.035)
**Paisley**	-0.006(0.015)	-0.595(0.614)	0.001(0.007)	-0.087(0.023)
**Perth**	-0.035(0.010)	-2.435(0.387)	0.008(0.005)	0.042(0.014)
**Galashiels**	-0.034(0.055)	-2.281(2.187)	-0.023(0.027)	0.111(0.081)
**Lerwick**	0.084(0.100)	1.077(3.958)	0.035(0.046)	-0.198(0.147)
